# Simulating rewetting events in intermittent rivers and ephemeral streams: A global analysis of leached nutrients and organic matter

**DOI:** 10.1111/gcb.14537

**Published:** 2019-02-25

**Authors:** Oleksandra Shumilova, Dominik Zak, Thibault Datry, Daniel von Schiller, Roland Corti, Arnaud Foulquier, Biel Obrador, Klement Tockner, Daniel C. Allan, Florian Altermatt, María Isabel Arce, Shai Arnon, Damien Banas, Andy Banegas‐Medina, Erin Beller, Melanie L. Blanchette, Juan F. Blanco‐Libreros, Joanna Blessing, Iola Gonçalves Boëchat, Kate Boersma, Michael T. Bogan, Núria Bonada, Nick R. Bond, Kate Brintrup, Andreas Bruder, Ryan Burrows, Tommaso Cancellario, Stephanie M. Carlson, Sophie Cauvy‐Fraunié, Núria Cid, Michael Danger, Bianca de Freitas Terra, Anna Maria De Girolamo, Ruben del Campo, Fiona Dyer, Arturo Elosegi, Emile Faye, Catherine Febria, Ricardo Figueroa, Brian Four, Mark O. Gessner, Pierre Gnohossou, Rosa Gómez Cerezo, Lluís Gomez‐Gener, Manuel A.S. Graça, Simone Guareschi, Björn Gücker, Jason L. Hwan, Skhumbuzo Kubheka, Simone Daniela Langhans, Catherine Leigh, Chelsea J. Little, Stefan Lorenz, Jonathan Marshall, Angus McIntosh, Clara Mendoza‐Lera, Elisabeth Irmgard Meyer, Marko Miliša, Musa C. Mlambo, Marcos Moleón, Peter Negus, Dev Niyogi, Athina Papatheodoulou, Isabel Pardo, Petr Paril, Vladimir Pešić, Pablo Rodriguez‐Lozano, Robert J. Rolls, Maria Mar Sanchez‐Montoya, Ana Savić, Alisha Steward, Rachel Stubbington, Amina Taleb, Ross Vander Vorste, Nathan Waltham, Annamaria Zoppini, Christiane Zarfl

**Affiliations:** ^1^ Leibniz‐Institute of Freshwater Ecology and Inland Fisheries (IGB) Berlin Germany; ^2^ Institute of Biology Freie Universität Berlin (FU) Berlin Germany; ^3^ Department of Civil Environmental and Mechanical Engineering Trento University Trento Italy; ^4^ Institute of Landscape Ecology and Site Evaluation University of Rostock Rostock Germany; ^5^ Department of Bioscience Aarhus University Silkeborg Denmark; ^6^ IRSTEA UR RIVERLY Centre de Lyon‐Villeurbanne Villeurbanne Cedex France; ^7^ Department of Plant Biology and Ecology Faculty of Science and Technology University of the Basque Country (UPV/EHU) Bilbao Spain; ^8^ Laboratoire d’Écologie Alpine (LECA) UMR CNRS‐UGA‐USMB 5553 Université Grenoble Alpes Grenoble France; ^9^ Department of Evolutionary Biology, Ecology and Environmental Sciences Faculty of Biology Biodiversity Research Institute (IRBIO) University of Barcelona Barcelona Spain; ^10^ Austrian Science Fund (FWF) Vienna Austria; ^11^ Department of Biology University of Oklahoma Norman Oklahoma; ^12^ Department of Evolutionary Biology and Environmental Studies University of Zurich Zürich Switzerland; ^13^ Centre of Edaphology and Applied Biology of Segura (CEBAS‐CSIC) Murcia Spain; ^14^ Zuckerberg Institute for Water Research The Jacob Blaustein Institutes for Desert Research Ben‐Gurion University of the Negev Beersheba Israel; ^15^ Université de Lorraine ‐ UR AFPA Vandoeuvre‐Les‐Nancy France; ^16^ Faculty of Environmental Science and EULA‐Chile Center Universidad de Concepción Concepción Chile; ^17^ Department of Geography University of California Berkeley California; ^18^ Mine Water and Environment Research Centre (MiWER) School of Science Edith Cowan University Perth Australia; ^19^ Instituto de Biología (ELICE‐RESTORES) Universidad de Antioquia Medellín Colombia; ^20^ Department of Environment and Science Queensland Government Brisbane Qld Australia; ^21^ Department of Geosciences Federal University of São João del‐Rei São João del‐Rei Brazil; ^22^ Department of Biology University of San Diego San Diego California; ^23^ School of Natural Resources and the Environment University of Arizona Tucson Arizona; ^24^ Grup de Recerca Freshwater Ecology, Hydrology and Management (FEHM) Departament de Biologia Evolutiva Ecologia i Ciències Ambientals Institut de Recerca de la Biodiversitat (IRBio) Universitat de Barcelona Barcelona Spain; ^25^ Centre for Freshwater Ecosystems La Trobe University Wodonga Vic. Australia; ^26^ Laboratory of Applied Microbiology University of Applied Sciences and Arts of Southern Switzerland Bellinzona Switzerland; ^27^ Australian Rivers Institute Griffith University Nathan Qld Australia; ^28^ Department of Environmental Biology Biodiversity Data Analytics and Environmental Quality Group University of Navarra Pamplona Spain; ^29^ Department of Environmental Science, Policy, and Management University of California Berkeley California; ^30^ LIEC Université de Lorraine Metz France; ^31^ Centro de Ciências Agrárias e Biológicas Universidade Estadual Vale do Acaraú Sobral Brazil; ^32^ Water Research Institute – National Research Council (IRSA‐CNR) Montelibretti (Rome) Italy; ^33^ Department of Ecology and Hydrology Regional Campus of International Excellence ‘Campus Mare Nostrum’ – University of Murcia Murcia Spain; ^34^ Institute for Applied Ecology University of Canberra Bruce Canberra ACT Australia; ^35^ Centre International de Recherche en Agronomie pour le Développement CIRAD UPR HortSys Montpellier France; ^36^ School of Biological Sciences University of Canterbury Christchurch New Zealand; ^37^ Great Lakes Institute for Environmental Research University of Windsor Windsor Canada; ^38^ INRA UAR 1275 DEPT EFPA Centre de recherche de Nancy Champenoux France; ^39^ Department of Ecology Berlin Institute of Technology (TU Berlin) Berlin Germany; ^40^ Faculté d'Agronomie Département d'Aménagement et de Gestion des Ressources Naturelles Université de Parakou Parakou Benin; ^41^ Department of Ecology and Environmental Science Umeå University Umeå Sweden; ^42^ MARE – Marine and Environmental Sciences Centre Department of Life Sciences University of Coimbra Coimbra Portugal; ^43^ Ezemvelo KZN Wildlife Pietermaritzburg South Africa; ^44^ Department of Zoology University of Otago Dunedin New Zealand; ^45^ BC3‐Basque Centre for Climate Change Leioa Spain; ^46^ ARC Centre of Excellence for Mathematical & Statistical Frontiers (ACEMS) and Institute for Future Environments School of Mathematical Sciences Queensland University of Technology Brisbane Qld Australia; ^47^ Department of Aquatic Ecology, Eawag The Swiss Federal Institute of Aquatic Science and Technology Dübendorf Switzerland; ^48^ Institute for Ecological Chemistry Plant Analysis and Stored Product Protection Julius‐Kuehn‐Institute Berlin Germany; ^49^ Department of Freshwater Conservation BTU Cottbus‐Senftenberg Bad Saarow Germany; ^50^ Department of Limnology Institute for Evolution and Biodiversity University of Münster Germany; ^51^ Department of Biology Faculty of Science University of Zagreb Zagreb Croatia; ^52^ Department of Freshwater Invertebrates Albany Museum Affiliated Research Institute of Rhodes University Grahamstown South Africa; ^53^ Department of Zoology University of Granada Granada Spain; ^54^ Missouri University of Science and Technology Rolla Missouri; ^55^ Terra Cypria – The Cyprus Conservation Foundation Limassol Cyprus; ^56^ Departamento de Ecología y Biología Animal Universidad de Vigo Vigo Spain; ^57^ Department of Botany and Zoology Faculty of Science Masaryk University Brno Czech Republic; ^58^ Department of Biology University of Montenegro Podgorica Montenegro; ^59^ School of Environmental and Rural Science University of New England Armidale NSW Australia; ^60^ Department of Biology and Ecology Faculty of Sciences and Mathematics University of Niš Niš Serbia; ^61^ School of Science and Technology Nottingham Trent University Nottingham UK; ^62^ Laboratoire d’Écologie et Gestion des Ecosystèmes Naturels (LECGEN) University of Tlemcen Tlemcen Algeria; ^63^ TropWATER (Centre for Tropical Water and Aquatic Ecosystem Research) College of Science and Engineering James Cook University Townsville Qld Australia; ^64^ Center for Applied Geosciences Eberhard Karls Universität Tübingen Tübingen Germany

**Keywords:** biofilms, leaching, leaf litter, rewetting, sediments, temporary rivers

## Abstract

Climate change and human pressures are changing the global distribution and the extent of intermittent rivers and ephemeral streams (IRES), which comprise half of the global river network area. IRES are characterized by periods of flow cessation, during which channel substrates accumulate and undergo physico‐chemical changes (preconditioning), and periods of flow resumption, when these substrates are rewetted and release pulses of dissolved nutrients and organic matter (OM). However, there are no estimates of the amounts and quality of leached substances, nor is there information on the underlying environmental constraints operating at the global scale. We experimentally simulated, under standard laboratory conditions, rewetting of leaves, riverbed sediments, and epilithic biofilms collected during the dry phase across 205 IRES from five major climate zones. We determined the amounts and qualitative characteristics of the leached nutrients and OM, and estimated their areal fluxes from riverbeds. In addition, we evaluated the variance in leachate characteristics in relation to selected environmental variables and substrate characteristics. We found that sediments, due to their large quantities within riverbeds, contribute most to the overall flux of dissolved substances during rewetting events (56%–98%), and that flux rates distinctly differ among climate zones. Dissolved organic carbon, phenolics, and nitrate contributed most to the areal fluxes. The largest amounts of leached substances were found in the continental climate zone, coinciding with the lowest potential bioavailability of the leached OM. The opposite pattern was found in the arid zone. Environmental variables expected to be modified under climate change (i.e. potential evapotranspiration, aridity, dry period duration, land use) were correlated with the amount of leached substances, with the strongest relationship found for sediments. These results show that the role of IRES should be accounted for in global biogeochemical cycles, especially because prevalence of IRES will increase due to increasing severity of drying events.

## Graphical abstract

In this study we experimentally simulated under laboratory conditions rewetting of leaves, riverbed sediments, and epilithic biofilms collected during the dry phase from intermittent rivers and ephemeral streams covering global spatial scale. We determined the amounts and quality of the leached nutrients and dissolved organic matter, assessed their inter‐substrate and cross‐climate differences, and estimated areal fluxes from 1 m^2^ of riverbeds. In addition, we evaluated the variance in leachate characteristics related to selected environmental variables and substrate characteristics.

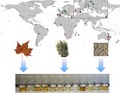

## 1 INTRODUCTION

Human activities and climate change cause global‐scale alterations in the flow regimes of rivers, which in turn are tightly linked to biogeochemical processes such as carbon processing (Arnell & Gosling, 2013; Bernhardt et al., 2018; Tonkin, Merritt, Olden, Reynolds, & Lytle, 2018). Currently, more than half of the global river network length is represented by intermittent rivers and ephemeral streams (IRES) – systems that cease to flow at some point in time and space (Acuña et al., 2014; Datry, Larned, & Tockner, 2014). Anthropogenic pressures alter the hydrological regime of perennial rivers toward intermittency, although the opposite can also happen at some locations. On the one hand, flow regulation, water diversion, groundwater extraction, and land‐use alteration promote the prevalence of river flow intermittence both spatially and temporally (Datry, Bonada, & Boulton, 2017; Pekel, Cottam, Gorelick, & Belward, 2016). On the other hand, naturally intermittent rivers turn permanent due to effluents from wastewater treatment plants or artificially enhanced discharge required for livestock and irrigation (Chiu, Leigh, Mazor, Cid, & Resh, 2017).

From a biogeochemical perspective, IRES function as punctuated biogeochemical reactors (Larned, Datry, Arscott, & Tockner, 2010; von Schiller, Bernal, Dahm, & Martí, 2017). During the dry phase, a diversity of substrates (leaf litter, epilithic biofilms, wood, animal carcasses, sediments) accumulate on the dry riverbed (Datry et al., 2018) . Absence of water reduces decomposition rates of substrates (for particulate organic matter, OM), while sunlight and intense desiccation alter their physico‐chemical properties, a process known as preconditioning (Abril, Muñoz, & Menéndez, 2016; Bruder, Chauvet, & Gessner, 2011; del Campo & Gómez, 2016; Dieter et al., 2011; Taylor & Bärlocher, 1996). When surface water returns after drying events, accumulated organic and inorganic substrates are rewetted and can be transported downstream (Corti & Datry, 2012; Obermann, Froebrich, Perrin, & Tournoud, 2007; Rosado, Morais, & Tockner, 2015). Rewetting during the so‐called “first flush events” also leads to massive pulsed releases of dissolved nutrients and dissolved organic matter (DOM; Arce, Sánchez‐Montoya, & Gómez, 2015; Gessner, 1991; von Schiller et al., 2011). Importantly, concentrations of the released substances may exceed baseflow values in perennial watercourses by several orders of magnitude and can thus substantially contribute to annual fluxes (Bernal, von Schiller, Sabater, & Marti, 2013; Corti & Datry, 2012; Skoulikidis & Amaxidis, 2009). Released nutrients and DOM fuel primary producers and heterotrophic organisms, alter nutrient and carbon cycling, and thus influence stream ecosystem metabolism (Austin et al., 2004; Baldwin & Mitchell, 2000; Fellman, Petrone, & Grierson, 2013; Jacobson & Jacobson, 2013; Skoulikidis, Vardakas, Amaxidis, & Michalopoulos, 2017). Furthermore, eutrophication and hypoxia can be a consequence of excess nutrient transport to downstream lakes, reservoirs, and coastal areas, where the mortality of fish and other aquatic organisms can increase (Bunn, Thoms, Hamilton, & Capon, 2006; Datry, Corti, Foulquier, Schiller, & Tockner, 2016; Hladyz, Watkins, Whitworth, & Baldwin, 2011; Whitworth, Baldwin, & Kerr, 2012).

Despite their widespread distribution and distinct role in biogeochemical cycling, IRES are notably missing in current analyses of global carbon budgets and other biogeochemical processes such as cycling of nutrients and DOM (Datry et al., 2018). Still, research on IRES is based primarily on studies spanning fine spatial extents (Leigh et al., 2016), which limits our understanding of their roles in ecosystem processes at the global scale (Datry et al., 2014; Skoulikidis, Sabater et al., 2017; von Schiller et al., 2017; but see Datry et al., 2018; Soria, Leigh, Datry, Bini, & Bonada, 2017). The contribution of IRES particularly to biogeochemical processes must be understood and quantified to correctly estimate carbon and nutrient fluxes. Studies indicating altered distribution of IRES in the future due to climate change (e.g. Milly, Dunne, & Vecchia, 2005) also emphasizes the need to adjust future river monitoring and conservation strategies.

The amounts and quality of dissolved compounds released from IRES upon rewetting, a process referred to as leaching (e.g. Gessner, 1991; Nykvist, 1963), depends primarily on the physico‐chemical characteristics and amounts of substrates accumulated on riverbeds. Leachates from leaf litter, the most abundant form of coarse particulate organic matter (CPOM) accumulated in dry riverbeds (Datry et al., 2018), are rich in dissolved organic carbon (DOC; up to 39% of the leaf bulk carbon content) including soluble sugars, carbonic and amino acids, phenolic substances, proteins, and inorganic nutrients (e.g., phosphorus, nitrogen, potassium; Bärlocher, 2005; Gessner, 1991; Harris, Silvester, Rees, Pengelly, & Puskar, 2016; Nykvist, 1963). Likewise, leaching from rewetted sediments of IRES releases large amounts of inorganic nitrogen (e.g. Arce, Sánchez‐Montoya, Vidal‐Abarca, Suárez, & Gómez, 2014; Merbt, Proia, Prosser, Casamayor, & von Schiller, 2016; Ostojic, Rosado, Miliša, Morais, & Tockner, 2013; Tzoraki, Nikolaidis, Amaxidis, & Skoulikidis, 2007). Furthermore, riverbeds can be covered by biofilm mats (hereafter referred to as “biofilm”), composed of microorganisms (algae, bacteria, fungi) embedded in a matrix of extracellular polymeric substances (Sabater, Timoner, Borrego, & Acuña, 2016), whose remnants can often be seen even during the dry phase. Biofilm's leachate may contain highly bioavailable organic carbon and nitrogen due to the accumulation of exudates and products of cell lysis (Romaní et al., 2017; Schimel, Balser, & Wallenstein, 2007). Physico‐chemical characteristics of substrates accumulated within IRES during the dry phase as well as the amounts of leached substances depend on environmental variables that act at both regional (climate influenced) and local scales (e.g. influenced by river geomorphology, land use, riparian canopy cover) (Aerts, 1997; Catalan, Obrador, Alomar, & Pretus, 2013; Datry et al., 2018; von Schiller et al., 2017).

The quantity and quality of dissolved substances leached from the channel beds of IRES during the rewetting process, and the environmental variables associated with variation in differences in leached amounts, has been little studied. However, such knowledge is essential for disentangling the role of IRES in biogeochemical processes under different scenarios of climate change. In the present study, we experimentally simulated pulsed rewetting events under controlled standardized laboratory conditions using substrates collected from 205 IRES located in 27 countries in five continents and covering five major climate zones. We aimed (a) to compare the amounts of nutrients and DOM, and the quality of DOM leached from leaf litter, biofilms, and bed sediments accumulated on dry IRES beds at the global scale as well as in different climate zones, (b) to explore and identify the environmental variables related to the variability in leached amounts, and (c) to estimate the potential area‐specific fluxes (per m^2^ of bed surface) of nutrients and OM leached during pulsed rewetting events. We focused on common nutrient and DOM species, which control essential ecosystem processes such as primary production and microbial respiration (Conley et al., 2009; Elser et al., 2007). Furthermore, we estimated the size categories and optical properties of released DOM as proxies of its quality.

Our first hypothesis was that in comparison with mineral substrates (sediments), leachates from organic substrates (biofilms and leaves) contain higher amounts of nutrients and DOM relative to the content of the respective element (carbon or nitrogen) in the substrate. In addition, substrates of organic origin also have a higher variability in the composition of leachates due to a higher species richness and compositional heterogeneity. Within our second hypothesis we expected that significant differences in the amounts of leached substances are observed among substrates sampled across different climate zones, with the highest amounts of nutrients and OM leached in the continental climate zone compared to others due to high litter quality (Boyero et al., 2017). In combination with the highest mass of litter observed (Datry et al., 2018) we expect this to result in the highest nutrient and OM fluxes from a representative area of dry river bed in the continental zone. Finally, we hypothesized that quantitative and qualitative composition of leachates will depend on substrate characteristics, which in turn are expected to correlate with environmental variables sampled at the study sites.

## 2 MATERIALS AND METHODS

### 2.1 Sampling sites, substrate collection, and environmental variables

**Figure 1:**
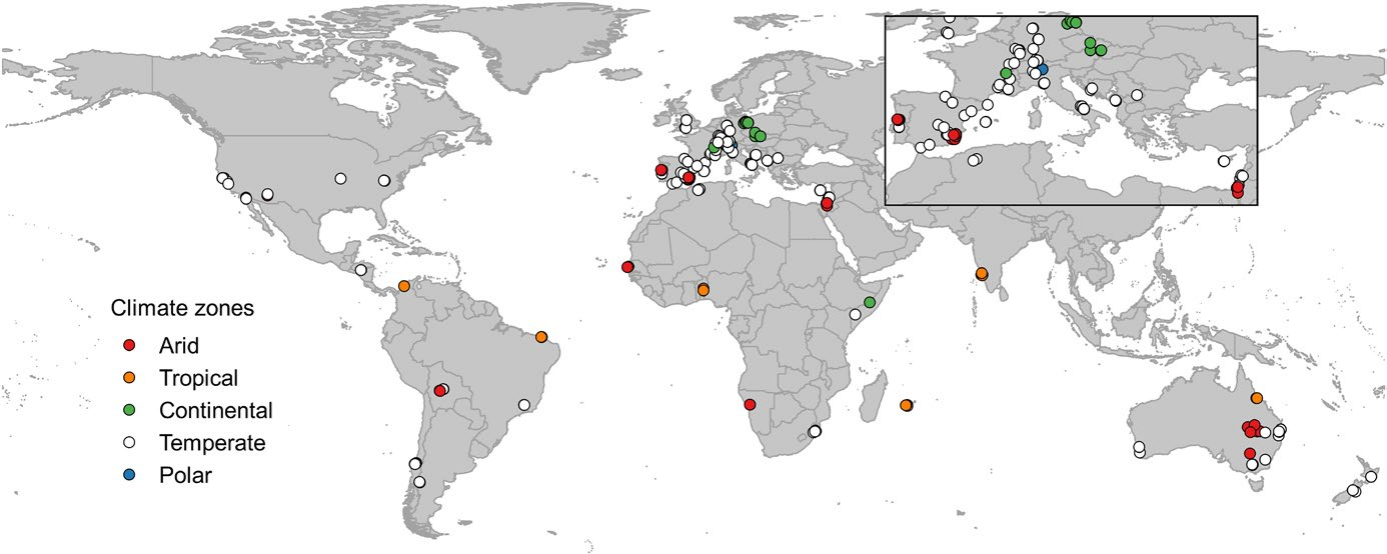
Location of the sampling sites (*N* = 205) across five climate zones. Climate zones according to Köppen–Geiger classes are marked with different colors [Colour figure can be viewed at http://www.wileyonlinelibrary.com/]

A total of 205 IRES, located in 27 countries and spanning five major Köppen–Geiger climate classes, were sampled during dry phases, following the standardized protocol of the 1,000 Intermittent Rivers Project (Datry et al., 2016, https://1000_intermittent_rivers_project.irstea.fr/, Figure 1). Five major climate zones were assigned to sites based on their location: arid (merging Köppen–Geiger classes BSh, BSk, BWh and BWk, *n* = 29), continental (Dfb, Dfc, *n* = 13), temperate (Cfa, Cfb, Csa, Csb, Cwa, *n* = 142), tropical (As, Aw, *n* = 19), and polar (ET, *n* = 1). Differences in sample size resulted from the occurrence of IRES and accessibility of sampling sites by researchers involved in the sampling campaign. A larger sample size increases the variability of the results while increasing the precision of the mean/median values, that is, reducing the variability of the sample mean/median. This needs to be considered in data evaluation and interpretation. For each river, one reach was selected and sampled for leaf litter (further referred as leaves), epilithic biofilms (biofilms), and sediments (details on material collection are provided in Supporting Information). After collection, field samples were further processed in the laboratory. Leaves and biofilms were oven‐dried (60°C, 12 hr) to achieve constant mass, reduce variability from fluctuations in water content (Boulton & Boon, 1991), and ensure cellular death of the leaf tissue. Oven‐drying mainly affects volatile and oxidizable compounds, which were not in the focus of our study. However, oven‐drying may increase the amount of leached substances from leaves and biofilms (e.g. Gessner & Schwoerbel, 1989). Bed sediments were sieved (2 mm) and air‐dried for 1 week. The dry material was placed in transparent plastic bags, shipped to laboratories responsible for further analyses (see Acknowledgements), and stored in a dry and dark room until processing and analysis.

Nine environmental variables were selected to analyze their association with leachate characteristics (Table 1). The variables were selected based on a conceptual understanding of the leaching process. As proxies of a regional‐scale influence, we used the aridity index and potential evapotranspiration (PET) extracted from the Global Aridity and PET database (for details see Datry et al., 2018). River width, riparian cover (%, visually estimated as the proportion of river reach covered by vegetation), dry period duration (estimated either with water loggers or repeated observations, precision: 2 weeks), altitude, and land cover (%) of pasture, forest, and urban areas within the catchment were selected as proxies of local influence. These local‐scale parameters (apart from land cover) were recorded in situ by participants of the 1,000 Intermittent Rivers Project. Land cover was derived using GIS maps. For details on the environmental variables sampled and substrate characteristics, see Table S1.

**Table 1:** Overview of the variables included in the partial least squares(PLS) regression models and transformations applied to meet assumptions of analysis

Variable	Description	Measurement units	Transformation	Variable in the PLS model
Environmental variables				
PET	Mean potential evapotranspiration for 1950–2000	mm/month	log(*x*)	X
Aridity	Mean annual aridity index for years 1950–2000	–	log(*x*)	X
Altitude	Altitude of the sampled reach	m above sea level	log(*x*)	X
Riparian cover	Percentage of the sampled reach covered by vegetation	%	log(*x* + 1)	X
Width of the sampled reach	Active channel width	m	log(*x*)	X
Dry period	Duration of the drying period	days	log(*x*)	X
Pasture cover	Percentage of pasture area within the river catchment	%	log(*x* + 1)	X
Forest cover	Percentage of forested area within the river catchment	%	log(*x* + 1)	X
Urban cover	Percentage of urban area within the river catchment	%	log(*x* + 1)	X
Chemical substrates characteristics				
% C	Carbon content	%	log(*x*)	X, Y
% N	Nitrogen content	%	log(*x*)	X, Y
C:N	Molar C:N ratio	–	log(*x*)	X, Y
Specific sediment characteristics				
Silt	Silt fraction	%	log(*x*)	X, Y
Sand	Sand fraction	%	log(*x*)	X, Y
Clay	Clay fraction	%	log(*x*)	X, Y
Mean size	Mean particle size	mm	log(*x*)	X, Y
Quantitative chemical characteristics of leachates				
DOC	Dissolved organic carbon	mg/g dry mass	log(*x*)	Y
DON	Dissolved organic nitrogen	mg/g dry mass	log(*x*)	Y
SRP	Soluble reactive phosphorous	mg/g dry mass	log(*x*)	Y
N‐NH_4_^+^	Ammonium	mg/g dry mass	log(*x*)	Y
N‐NO_3_^−^	Nitrate	mg/g dry mass	log(*x*)	Y
Qualitative chemical characteristics of leachates				
SUVA_254_	Specific ultraviolet absorbance	mg C/L	–	Y
FI	Fluorescence index	–	log(*x* + 1)	Y
HIX	Humification index	–	log(*x* + 1)	Y
*β*:*α*	Ratio of autochthonous to allochtonous dissolved organic matter	–	log(*x* + 1)	Y
DOC:DON	Ratio of DOC to DON concentration	–		Y
Phenolics:DOC	Ratio of phenolics to DOC concentration	–	log(*x* + 1)	Y
LMWS	Low molecular weight substances	%		Y
BP	Biopolymers	%		Y
HS	Humic substances	%		Y

### 2.2 Leaching experiments

Rewetting was simulated in the laboratory by exposing dried substrates to leaching solutions as a proxy for their exposure in situ to river water during first flush events. Leaves were cut into approximately 0.5 cm × 0.5 cm pieces and homogenized in glass beakers using a spoon. If the sample contained conifer‐needles (approximately 30% of samples), these were cut into fragments of approximately 4 ± 0.5 cm length. From each sample, 0.5 ± 0.01 g were weighed, put into 250 ml dark glass bottles and filled with 200 ml of a 200 mg/L NaCl leaching solution to mimic ionic strength of the stream water and thus to avoid extreme osmotic stress on microorganisms’ cells upon rewetting (e.g. McNamara & Leff, 2004). For biofilms, sub‐samples homogenized as previously described were weighed to 1 ± 0.01 g, and placed in dark glass bottles filled with 100 ml of the leaching solution. Sediment samples (20–60 g) were homogenized in the same way, weighed to 10 ± 0.1 g, transferred into 250 ml dark glass bottles, and filled with 100 ml of the leaching solution. The selected mass of each substrate in relation to the volume of leaching solution aimed on maximizing the leaching yield by avoiding high concentrations of dissolved substances that could lead to saturation so that substances cannot dissolve further.

Preliminary investigations of the effect of temperature and time on leaching (tested at temperatures of 4 and 20°C and leaching durations of 4 and 24 hr, corresponding to temperatures and durations most commonly applied in leaching studies due to the rapid nature of the leaching process, data not shown), indicated selection of a constant temperature of 20°C and leaching duration of 4 hr. The selected duration reflects the time when most of the dissolved substances are leached and minimizes microbial modification of leachates upon rewetting. Bottles containing substrates and the leaching solution were capped and placed on shaking tables (100 rpm) in a climate chamber in darkness. Two subsamples (technical replicates) of each substrate type from each sampling site were leached whenever enough material was available (70% of the samples). Otherwise a single technical replicate was used.

After 4 hr, the leachate from the bottle was filtered through 8.0 µm cellulose acetate and 0.45 µm cellulose nitrate membrane filters (both Sartorius, AG Göttingen, Germany) which were prerinsed with 1 L of de‐ionized water per filter, using a vacuum pump. Filtered leachates were collected in 200 ml glass flasks prerinsed with 50 ml of the filtered leachate. If sufficient substrate was available, two subsamples were leached to cover possible heterogeneity of substrate composition, but combined later in one glass flask to have one representative composite sample for further analysis. Leachates were then transferred into HCl prewashed 25 ml plastic bottles prior to further chemical analyses (see details in Supporting Information).

### 2.3 Physical and chemical characterization of substrates and leachates

Organic carbon (C) and total nitrogen (N) content of substrates (%C and %N, respectively) were determined using elemental analyzers (for details see Supporting Information). Sediment texture descriptors (fractions [%] of sand, silt, clay, and their mean and median particle size) were determined with a laser‐light diffraction instrument (see Supporting Information).

Using standard analytical methods (for details see Supporting Information) we analyzed the following substances in leachates: DOC, soluble reactive phosphorus (SRP), ammonium (N‐NH_4_^+^), nitrate (N‐NO_3_^−^), and phenolics.

The concentration of nutrients and OM in leachates was used to calculate leached amounts per gram of dry substrate (total leached amounts) and per gram of the respective element, C or N, in the substrate (relative leached amounts). Areal fluxes upon rewetting were calculated from total leached amounts and mass of substrate accumulated in the field.

### 2.4 Characterization of DOM quality

To determine concentrations of dissolved organic nitrogen (DON) and the composition of DOM based on size categories, we used size‐exclusion chromatography with organic carbon and organic nitrogen detection (LC‐OCD‐OND analyzer, DOC‐Labor Huber, Karlsruhe, Germany) (details are provided in Supporting Information). A subset of leaves, biofilms, and sediments sampled from 77 rivers was selected randomly to cover all climate zones. We selected limited samples due to the time‐consuming nature of this analysis (2.5 hr per sample). Leachates produced from these substrates (as described previously) were selected for further analysis, in cases where concentrations of DOC in leachates did not exceed the measuring limits of the chromatograph (the final set included leachates from 52 leaf, 11 biofilm, and 77 sediment samples). We classified DOM into three major sub‐categories: (a) biopolymers (BP), (b) humic or humic‐like substances (HS) including building blocks (HS‐like material of lower molecular weight), and (c) low molecular‐weight substances (LMWS). The concentration of each category was normalized to the total DOC concentration, and is thus given as the fraction (%) of the total DOC.

To obtain indices of DOM quality (for details see Fellman, Hood, & Spencer, 2010; Hansen et al., 2016), we simultaneously determined absorbance spectra of DOM and fluorescence excitation‐emission matrices (EEM) using a spectrofluorometer (Horiba Jobin Yvon Aqualog; Horiba Scientific Ltd, Kyoto, Japan). Specific UV absorbance values were calculated at a wavelength of 254 nm (SUVA_254_), which are correlated with aromatic carbon content (Weishaar et al., 2003), by dividing decadal absorbance by DOC concentration (mg C/L) and cuvette length (m). The fluorescence index (FI), humification index (HIX), and freshness index (*β*:*α*) were calculated from fluorescence EEM for all DOM samples (for details see Supporting Information). The FI indicates whether DOM is derived from terrestrial sources (e.g. plant or soil, FI value ~1.4) or microbial sources (e.g. extracellular release, leachates from bacterial and algal cells lysis, FI value ~1.9) (McKnight et al., 2001). The HIX indicates the extent of DOM humification (degradation) (Ohno, 2002; Zsolnay, Baigar, Jimenez, Steinweg, & Saccomandi, 1999), with HIX <0.9 indicating DOM derived from relatively recent (plant and algae) inputs (Hansen et al., 2016). The freshness index, that is, the ratio of autochthonous (*β*) vs. allochthonous (*α*) DOM, indicates dominance by recently produced or decomposed DOM (values ~0.6–0.7 indicate more decomposed allochtonous DOM; Parlanti, Worz, Geoffroy, & Lamotte, 2000; Wilson & Xenopoulos, 2008). EEM were corrected for Raman scatter, Rayleigh, and inner filter effects before calculation of the fluorescence indices (Mcknight et al., 2001; Parlanti et al., 2000).

### 2.5 Calculation of the total areal flux of nutrients and OM

Total areal flux of nutrients and OM per square meter of the riverbed was calculated based on information about the mass of leaves and biofilm accumulated on the dry riverbeds (Datry et al., 2018), as well as on average mass of sediment per square meter of surface area. For the latter, we assumed an average density of sediments of 1.6 g/cm^3^ (Hillel, 1980) and the depth of the sediments potentially affected by a rewetting event to be 10 cm (see Merbt et al., 2016), which also corresponds to the depth of the sampled sediment layer according to the sampling protocol. We acknowledge that this assumption should be considered with caution as high variability in sediment densities can be found in nature (e.g. Boix‐Fayos et al., 2015) and contribution of sediment layers within 10 cm depth to leaching also may differ (e.g. Merbt et al., 2016).

Overall, the total areal flux is the sum of nutrients and OM leached from all substrates found within the dry riverbed. To execute a global comparison of total areal fluxes, samples from 157 reaches were selected for which a complete set of nutrients and OM concentrations (except DON) were available. Reaches for which one or more chemical measurements were identified as technical outliers after exploration with boxplots and Cleveland dotplots (Zuur, Ieno, & Elphick, 2010) were excluded. We assume these calculations reflect spatial differences in surface fluxes of nutrients and OM across a range of sampled IRES.

### 2.6 Statistical analyses

Differences in the total and relative leached amounts of nutrients and DOM from different substrates (Hypothesis 1), as well as between substrates collected in different climate zones and estimated fluxes from different climate zones (Hypothesis 2), were assessed using Kruskal–Wallis nonparametric tests followed by Dunn's tests with Bonferroni correction for post‐hoc comparisons. The level of significance was set to 0.0167 to account for multiple comparisons among the three substrates and to 0.0083 to account for comparisons among the four main climate zones (calculated as 0.05/[*k*(*k*−1)/2], where *k* is the number of groups) (Dunn, 1964). The polar climate zone was excluded from the comparison as there was only one sampling location in this category. Biofilm leachates were excluded from the cross‐climate comparison as the majority of samples were taken in the temperate zone (35 out of 41 samples). Variability in leached amounts (Hypothesis 1) was assessed based on interquartile difference (quartile three of data distribution minus quartile one) expressed in percentages. This measure of variability accounts for differences in data distributions of nutrients and DOM amounts leached from different substrates and facilitates comparison.

In order to identify the environmental variables and substrate characteristics driving the quantitative (amounts of nutrients and OM) and qualitative (DOM quality) characteristics of the leachates partial least squares (PLS) regression models were applied (Wold, Sjöstrom, & Eriksson, 2001). This approach allows exploration of the relationship between collinear data in matrices *X* (independent variable) and *Y* (dependent variable). An overview of the components to be included in the models is given in Table 1. Performance of the model is expressed by *R* ^2^ *Y* (explained variance). The influence of every *X* variable on the *Y* variable across the extracted PLS components (latent vectors that explain as much as possible of the covariance between *X* and *Y*) is summarized by the variable influence on projection (VIP) score (Table 3). The VIP scores of every model term (*X*‐variables) are cumulative across components and weighted according to the amount of *Y*‐variance explained in each component (Eriksson, Johansson, Kettaneh‐Wold, & Wold, 2006). *X*‐variables with VIP > 1 are most influential on the *Y*‐variable, while variables with 1 > VIP > 0.8 are moderately influential. Values negatively correlated with the *Y*‐variable were multiplied by a coefficient of negative one to facilitate interpretation. Data were transformed prior to analyses to meet the assumptions of normal distribution and homoscedasticity (Table 1).

**Figure 2:**
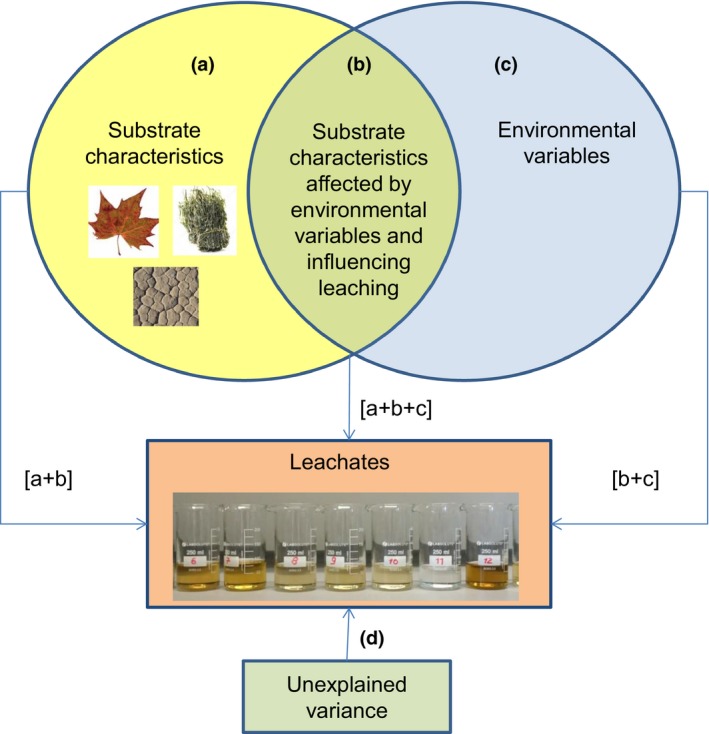
Variance partitioning among variables that influence leaching of nutrients and organic matter from substrates accumulated in intermittent rivers and ephemeral streams. * Fraction a – variance explained by the substrate characteristics; fraction b – variance explained by the effect of environmental variables on substrate characteristics measured in the study; fraction c – variance explained by the environmental variables; [d] – unexplained variance. ** [a + b] – effect of the substrate characteristics on leachate characteristics; [b + c] – effect of the environmental variables on leachate characteristics; [a + b + c] – effect of the environmental variables on leachate characteristics through their impact on substrate characteristics. [Colour figure can be viewed at http://www.wileyonlinelibrary.com/]

In order to partition the variance in quantitative and qualitative characteristics of nutrients and DOM explained by different groups of variables (environmental variables, substrate characteristics, and the effect of environmental variables through their effect on measured substrate characteristics), we used the approach suggested in Borcard, Legendre, and Drapeau (1992) (Figure 2). The following PLS‐regression models were run to distinguish fractions of explained variance in the quantitative/qualitative characteristics of the leachates:

‐ Fraction [a + b] – explained by substrate characteristics;
‐ Fraction [b + c] – explained by environmental variables;
‐ Fraction [a + b + c] – explained by environmental variables and measured substrate characteristics.

From each PLS‐regression model, the explained variance *R* ^2^ *Y* was calculated and used to calculate the fraction of variance explained by each set of predictors separately (Borcard et al., 1992). For the PLS regression analysis, we selected the complete set of variables for which the required data (all predictors and response variables, Table 1) were available. We ran partitioning of variance for the set of samples on the global scale and individually for each climate zone. For biofilms, the analysis was done for samples of the temperate zone only because of the limited number of samples from other climate zones.

All statistical analyses were performed in r 3.2.2 (R Core Team, 2017), except for the PLS analysis which was conducted using xlstat software (XLSTAT, 2017, Addinsoft, Germany).

## 3 RESULTS

### 3.1 Leached amounts of nutrients and DOM species

#### 3.1.1 Total and relative leaching rates

**Figure 3:**
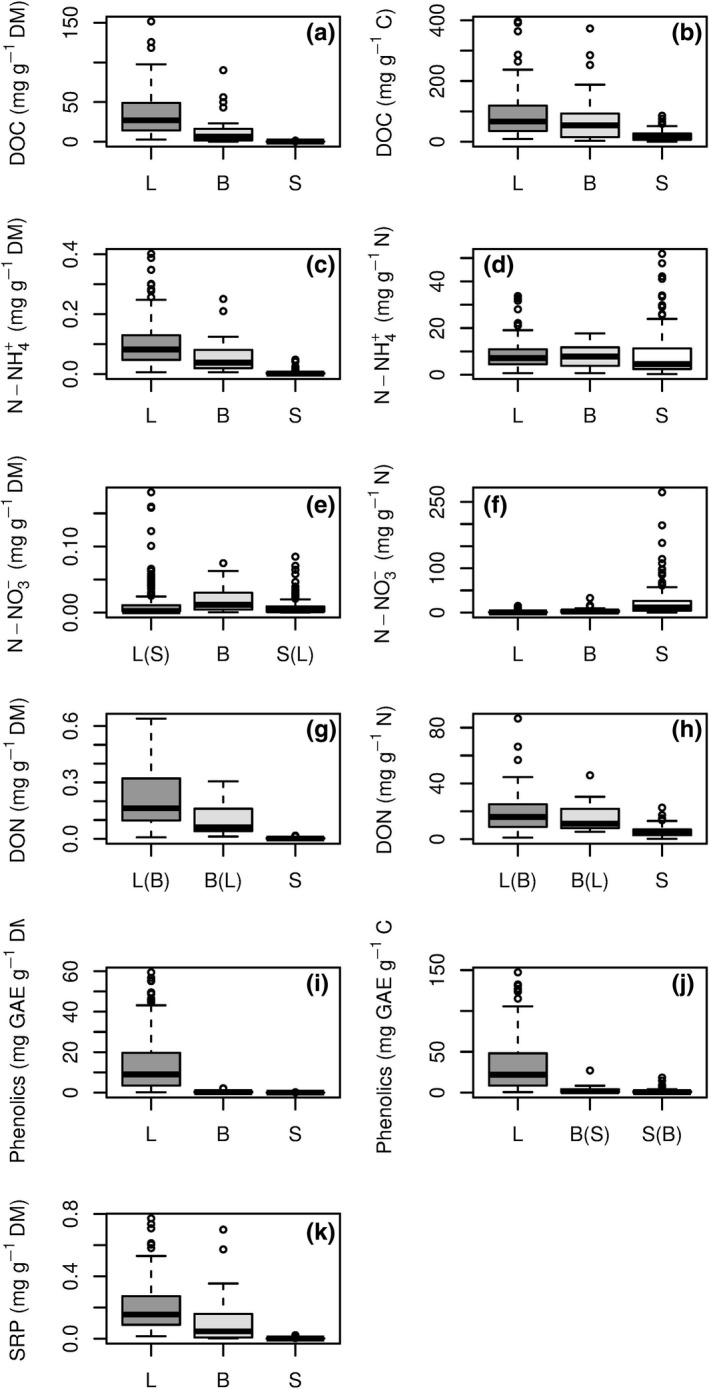
Total (left) and relative (right) leached amounts of nutrients and dissolved organic matter from leaves (L), biofilms (B), and sediments (S) of IRES globally. Box: median, interquartile range (25%–75%), and outliers (i.e. values that exceed 1.5 interquartile range). DM – dry mass; GAE – gallic acid equivalent. Note: Relative leached amounts of SRP were not estimated. For parameter acronyms see Table 1. Letters in parentheses on the *x*‐axis indicate nonsignificant difference between leachates from specified substrates (*p* > 0.0167, Dunn test for post‐hoc comparison; see Section 2)

The total leached amounts (mg/g dry mass) of nutrients (except N‐NO_3_^−^) and DOM were highest for leaves, followed by biofilms, and sediments (Figure 3; Table S2). The leached amounts of N‐NO_3_^−^ were highest for biofilms (Kruskal–Wallis test, *χ* ^2^ = 15.8, *df* = 2, *p* < 0.0001; Dunn's test for multiple comparison, *p* < 0.0001), and no significant difference was found between leaves and sediments (Dunn's test, *p* = 0.3). Leached amounts of DON from leaves and biofilms were not significantly different (Kruskal–Wallis test, *χ* ^2^ = 105.7, *df* = 2, *p* < 0.0001; Dunn's test, *p* = 0.2).

The total leached amounts of nutrients and DOM from leaves and biofilms decreased in a similar sequence: DOC > phenolics > DON > SRP > N‐NH_4_^+^ > N‐NO_3_^− ^(based on median values). The total leached amounts from sediments decreased in the following order: DOC > phenolics > N‐NO_3_^−^ > N‐NH_4_^+ ^ ≈ DON > SRP (Table S2).

The relative leached amounts of DOC and phenolics (mg/g C) and DON (mg/g N) were highest for leaves, followed by biofilms and sediments (Figure 3; Table S2). However, there were no significant differences for the amounts of DON between leaves and biofilm leachates (Kruskal–Wallis test, *χ* ^2^ = 51.6, *df* = 2, *p* < 0.0001; Dunn's test, *p* = 0.8), nor for phenolics between biofilms and sediments (Kruskal–Wallis test, *χ* ^2^ = 265.4, *df* = 2, *p* < 0.0001; Dunn's test, *p* = 0.2). Relative leached amounts of N‐NH_4_^+^ were highest for biofilms, followed by leaves and bed sediments, with a significant difference between leaves and sediments (Kruskal–Wallis test, *χ* ^2^ = 265.4, *df* = 2, *p* < 0.0001; Dunn's test, *p* < 0.001). For N‐NO_3_^−^, relative leached amounts decreased significantly from sediments to biofilms and leaves (Kruskal–Wallis test, *χ* ^2^ = 204.4, *df* = 2, *p* < 0.0001; Dunn's test, *p* < 0.001; Figure 3; Table S2).

For all substrates, we observed large variations in the total and relative leached amounts of nutrients and DOM (Figure 3, Table S2). The highest variability in total and relative leached amounts of DOC, N‐NO_3_^−^, and SRP was observed for biofilms, which was up to 10 times higher than for sediments and leaves. Sediments had the highest variability in the total leached amounts of DON and relative leached amounts of N‐NH_4_^+^ and phenolics. For leaves, the highest variability was found in the relative leached amounts of DON.

### 3.2 Qualitative DOM characterization

**Figure 4:**
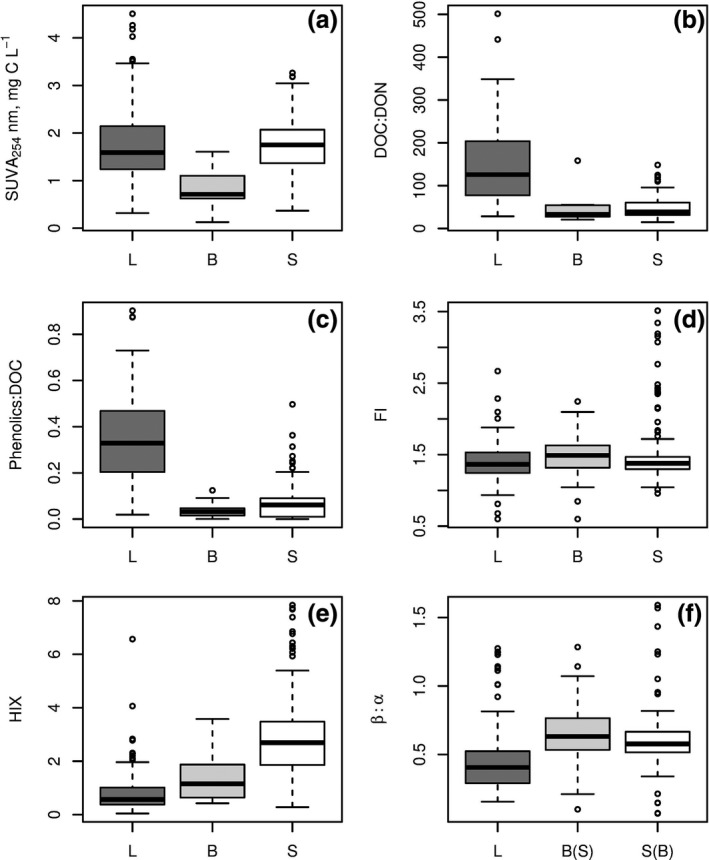
Qualitative characteristics of dissolved organic matter leached from leaves (L), biofilms (B), and sediments (S) of IRES globally. Box: median, interquartile range (25%–75%), and outliers (i.e. values that exceed 1.5 interquartile range). For parameter acronyms see Table 1. Letters in parentheses on the *x*‐axis indicate that the difference between leachates from specified substrates was nonsignificant (*p* > 0.0167, Dunn test for post‐hoc comparison; see Section 2)

Values of SUVA_254_, a proxy for aromatic carbon content, decreased from sediments and leaves to biofilms, with no significant difference between sediments and leaves (Kruskal–Wallis test, *χ* ^2^ = 55.8, *df* = 2, *p* < 0.0001; Dunn's test, *p* = 0.4) (Figure 4; Table S3).

Ratios of DOC:DON and phenolics:DOC were highest in leachates from leaves, while differences between sediments and biofilms were not statistically significant (Dunn's test following a Kruskal–Wallis test, *p* = 0.8 and *p* = 0.06 respectively; Table S3).

The *β*:*α* ratio indicated a prevalence of allochthonous DOM in leachates from all substrates. The proportion of allochthonous DOM was highest in leachates from biofilms, followed by sediments, then leaves, but there was no significant difference between biofilms and sediments (Kruskal–Wallis test, *χ* ^2^ = 197.4, *df* = 2, *p* < 0.0001; Dunn's test, *p* = 0.4). The degree of DOM humification based on HIX values was highest for sediments followed by biofilms and leaves, with statistically significant differences among all substrates (Kruskal–Wallis test, *χ* ^2^ = 96.94, *df* = 2, *p* < 0.0001; Dunn's tests <0.0001). Values of FI indicated the presence of OM derived from terrestrial sources in all leachates, with no significant differences among substrates (Kruskal–Wallis test, *χ* ^2^ = 6.3, *df* = 2, *p* = 0.043).

**Figure 5:**
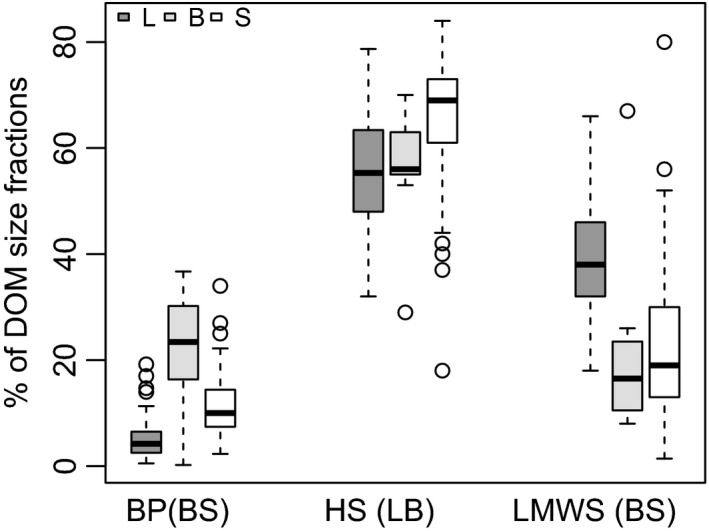
Size fractions of dissolved organic matter (DOM) leached from leaves (L), biofilms (B), and sediments (S) of IRES globally. BP, biopolymers; HS, humic substances; LMWS, low molecular weight substances. Box: median, interquartile range (25%–75%), and outliers (i.e. values that exceed 1.5 interquartile range). Letters in parentheses on the *x*‐axis indicate that the difference between leachates from specified substrates was nonsignificant (*p* > 0.0167, Dunn test for post‐hoc comparison; see Section 2)

In all leachates, HS was the dominant fraction of DOM followed by BP and LMWS (Figure 5; Table S3). The highest proportion of HS in DOM was in sediment leachates, while between leachates of leaves and biofilms the percentage of HS did not significantly differ (Kruskal–Wallis test, *χ* ^2^ = 29.9, *df* = 2, *p* < 0.0001; Dunn's test, *p* = 0.9). The highest percentage of LMWS was present in leaf leachates with the median twice as high as in sediments and biofilms. The highest percentage of BP was found in leachates from biofilms with the median values two and six times higher than in sediments and leaves, respectively. For LMWS and BP, the difference between biofilms and sediments was not statistically significant (Dunn's test following a Kruskal–Wallis test, *p* = 0.7 and *p* = 0.06 respectively).

### 3.3 Differences in amounts of leached substances and DOM quality across climate zones

Cross‐climate differences in amounts of leached substances and qualitative characteristics of DOM depended on the type of substrate (Table 2; Table S4). For leaves, a significant difference in the total leached amounts was observed only for N‐NH_4_^+^ between continental and arid zones, as well as between continental and temperate zones (Dunn post‐hoc tests following a Kruskal–Wallis test, *p* < 0.0001, Table S4). All variables measured in leaves showed highest concentration in the continental zone, except for N‐NO_3_^−^ (highest in the tropical zone) and DON (highest in the arid zone). For sediments, significant differences in leached amounts were found for all variables except phenolics (Kruskal–Wallis test, *χ* ^2^ = 5.43, *df* = 3, *p* = 0.143). In all cases, the highest total leached amounts were found in samples from the continental zone and the lowest in leachates from the arid zone (Table 2; Table S4). Leached amounts of nutrients and DOM from leaves and sediments from the temperate zone, the most commonly sampled zone in the study, followed leached amounts found in the tropical zone, however, with no significant difference (Table 2; Table S4). The relative leached amounts did not differ significantly among climate zones for leaves or sediments (Table S4).

**Table 2:** Total and relative leaching rates of nutrients and organic matter species from leaves and bed sediments of IRES (median). For abbreviations, see Table 1

Parameter	Unit	Leaching rate	Leaves				Sediments			
			Arid	Continental	Temperate	Tropical	Arid	Continental	Temperate	Tropical
DOC	mg/g dry mass	Total	30.98	47.40	25.30	22.90	0.06	0.25	0.07	0.08
	mg/g C	Relative	86.28	108.86	58.10	66.50	14.66	13.30	12.24	19.92
N‐NH_4_^+^	mg/g dry mass	Total	0.06	0.14	0.08	0.105	0.001	0.004	0.0015	0.002
	mg/g N	Relative	7.80	11.70	6.60	8.20	6.01	4.30	4.51	6.36
N‐NO_3_^−^	mg/g dry mass	Total	0.004	0.006	0.002	0.008	0.003	0.01	0.004	0.005
	mg/g N	Relative	0.43	0.32	0.27	0.59	13.03	10.57	10.48	18.32
DON	mg/g dry mass	Total	0.30	0.22	0.14	0.29	0.001	0.007	0.002	0.002
	mg/g N	Relative	22.03	17.80	12.50	28.80	6.10	4.90	4.80	2.30
SRP	mg/g dry mass	Total	0.11	0.24	0.15	0.16	0.0004	0.002	0.0005	0.0007
Phenolics	mg of GAE/g of substrate	Total	9.08	20.18	8.38	8.92	0.003	0.010	0.005	0.007
	mg of GAE/g of C	Relative	0.23	0.51	0.20	0.24	0.008	0.006	0.005	0.009
SUVA_254_	mg C/L		1.60	1.44	1.57	1.88	1.21	2.01	1.75	1.78

Note

GAE: gallic acid equivalent.

Aromatic carbon content (a proxy used to access cross‐climate differences in bioavailability) leached from leaves was not significantly different among climate zones (Kruskal–Wallis test, *χ* ^2^ = 3.82, *df* = 3, *p* = 0.28). For sediments, a statistically significant difference was found between samples from the arid and the continental zone (Dunn's test, *p* = 0.003; Table S4), with leachates from the arid zone having lower aromaticity.

### 3.4 Effects of environmental variables and substrate characteristics

#### 3.4.1 Effects on the amounts of leached nutrients and DOM

**Figure 6:**
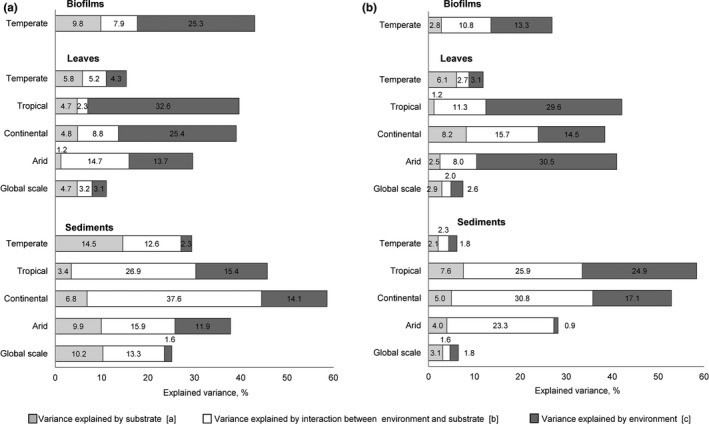
Partitioning of variance in quantitative composition (a) and qualitative characteristics (b) of leachates on global and regional scales (values indicate percentage of variance (*R* ^2^ *Y*) explained). Note: For biofilms, the analysis was done on data from the temperate zone only because of the limited amount of samples from other climate zones

On a global scale, 25% of the variance in the amounts of nutrients and DOM leached from sediments could be explained by selected variables (fraction [a + b + c]), which was more than twice that for leaves (11%) (Figure 6a,b). For sediments, around 23% of the variance could be explained by the effect of substrate characteristics (fraction [a + b]), around 15% by the effect of environmental variables (fraction [b + c]), and 13% by the effect of environmental variables on substrate characteristics (fraction [b]) (Figure 6a). For leaves, the substrate characteristics and the environmental variables explained approximately an equal percentage of variance, 8% and 6% respectively, which was much lower than that explained for sediments. Environmental variables and substrate characteristics accounted for 3% of variance in the quantitative composition of leaf leachates. For both substrates, the most influential variables (VIP >1) were C fraction, N fraction, PET, and in the case of leaves, C:N and pasture cover within the river catchment (Table 3).

**Table 3:** Ranking of environmental variables and substrates characteristics that explain variance in quantitative composition (A) and qualitative characteristics (B) of leachates at global and regional scales according to their value of VIP (variable influence on projection) in the PLS analysis. VIP > 1 indicate highly influential predictors (dark grey), 1 > VIP > 0.8 indicate moderately influential variables (medium grey), VIP < 0.8 – variables of low influence (light grey)

Predictors	Sediments					Leaves					Biofilms
	Global (170)	Arid (20)	Cont.(10)	Temp. (125)	Trop. (15)	Global (183)	Arid (21)	Cont. (13)	Temp. (131)	Trop. (18)	Temp. (23)
**(A) Quantitative composition of leachates**											
PET	1.445	0.111	0.557	1.441	1.367	1.129	0.776	1.352	1.134	1.180	0.833
Aridity	0.371	1.444	0.388	0.303	0.708	0.765	0.979	1.371	0.505	1.844	1.131
Dry period	0.495	0.580	1.767	0.325	1.061	0.630	0.745	0.706	0.752	1.000	0.534
River width	0.867	0.920	1.095	0.868	0.333	0.821	0.683	1.207	0.950	0.938	0.852
Riparian cover	0.955	1.243	0.805	0.765	0.394	0.744	0.869	0.702	0.567	0.554	0.829
% pasture	0.153	0.506	0.727	0.205	0.063	1.225	1.397	0.442	1.160	1.467	0.189
% forest	0.445	0.264	1.030	0.495	0.472	0.528	1.139	0.871	0.815	0.776	0.439
% urban	0.389	0.073	0.929	0.532	1.030	0.163	0.674	1.116	0.360	0.865	0.558
Altitude	0.784	0.731	0.547	0.630	0.881	0.549	1.170	1.268	0.982	0.439	1.041
%C	1.768	1.390	0.889	1.782	1.170	1.132	0.990	0.365	1.454	0.668	1.424
% N	2.062	1.657	1.345	2.117	1.000	1.673	1.510	0.933	1.279	0.705	2.026
C:N	0.336	0.897	0.509	0.238	1.761	1.526	0.576	1.017	1.348	0.618	0.757
% sand	0.897	1.368	1.100	0.856	0.986						
% silt	0.960	0.744	1.139	1.056	1.177						
% clay	0.920	1.055	1.145	1.003	1.159						
Mean size	0.902	1.136	1.067	0.923	1.004						
**Var explained %**	**25.1**	**37.8**	**58.6**	**29.4**	**45.7**	**11.1**	**29.6**	**37.5**	**15.3**	**34.2**	**47.5**
**(B) Qualitative characteristics of leachates**											
PET	1.100	0.582	0.903	0.377	1.734	0.496	1.696	1.097	0.601	1.378	1.538
Aridity	0.432	0.526	1.180	0.430	1.217	0.680	1.074	0.983	0.853	1.167	0.703
Dry period	0.468	0.704	1.141	0.555	0.877	0.613	1.555	1.224	0.599	1.786	0.690
River width	0.864	0.841	0.375	1.230	0.281	1.027	0.255	0.438	1.045	0.934	0.497
Riparian cover	0.786	0.645	1.092	0.265	0.234	0.452	0.638	1.093	0.176	0.516	0.564
% pasture	0.589	0.217	1.257	0.988	0.310	0.716	0.794	0.722	0.652	0.728	1.081
% forest	0.942	1.655	1.227	0.802	0.929	0.585	0.972	0.640	0.752	0.564	1.140
% urban	0.469	0.478	0.095	0.108	1.161	1.097	0.860	0.712	0.385	1.128	1.235
Altitude	1.124	0.191	1.094	1.386	0.683	1.104	0.722	1.002	1.059	0.369	0.869
%C	1.148	1.553	0.577	0.562	0.882	2.311	0.824	0.516	2.329	0.243	1.057
% N	0.688	1.059	0.575	0.729	0.878	0.822	0.846	1.311	1.036	1.130	1.165
C:N	0.792	0.812	1.108	0.939	1.381	0.600	0.921	1.587	0.820	0.905	0.937
% sand	1.379	1.609	1.080	1.309	0.935						
% silt	1.443	1.201	1.222	1.564	1.119						
% clay	1.403	0.967	1.164	1.492	1.161						
Mean size	1.389	1.247	0.979	1.455	0.952						
**Var explained %**	**6.4**	**28.2**	**52.9**	**6.2**	**58.9**	**7.5**	**41.1**	**38.7**	**11.9**	**42.2**	**26.9**

For both sediments and leaves, the highest percentage of variance in amounts of leached nutrients and DOM was explained for the continental and tropical zones (59% and 46% for sediments, 39% and 40% for leaves respectively, Figure 6a). Substances leached from sediments from these regions were explained mostly by the environmental variables and their effect on substrate characteristics. High VIP was found for the dry period duration, N fraction and textural classes (both zones), river width and forest cover (continental), PET, urban cover, and fraction of C (tropical). In contrast, for leaves in these zones, most of the variance was explained by environmental variables alone and not by their effect on the substrates. Environmental variables with high VIP in these zones were PET and aridity (in both), river width and altitude (in the continental zone), as well as pasture cover and dry period duration (in the tropical zone) (Table 3).

For the temperate zone, the results of variance partitioning were available for all analyzed substrates. Here, the total variance in leachates was best explained for biofilms (48%) followed by sediments (30%) and leaves (15%). In contrast to sediments and leaves, the variance in biofilm leachates was better explained by environmental variables (VIP >1 for aridity and altitude) than by substrate characteristics.

### 3.5 Effects on qualitative characteristics of DOM

For sediments and leaves, the percentage of variance that was explained for qualitative characteristics of DOM on the global‐scale was much lower (around 7% for each of the substrates) than that for the amounts of leached substances (Figure 6b). The contribution of environmental variables, substrate characteristics, and effect of environmental variables on substrate characteristics to the total variance was approximately equal (Figure 6). Influential variables with VIP >1 were altitude and C fraction (for both substrates), PET and texture (for sediments), and river width and urban cover (for leaves).

For sediments, as in the case of amounts of leached substances, the variance across sampling sites was explained best in the tropical (58%) and continental (53%) zones, and was driven mainly by the environmental variables and their effect on substrate characteristics. Variables with VIP >1 in both zones were sediment texture (fraction of silt and clay) and, additionally PET, aridity, and urban cover in samples from the tropical zone, and pasture and forest cover, riparian cover, aridity, and dry period duration in samples from the continental zone (Table 3). For sediments in the arid zone, the explained variance was around 28% and the share of groups of variables that explained the observed variance was different. In particular, almost all variance explained by environmental variables was due to the effect of environmental variables on substrates (VIP >1 for texture, %C, %N, and forest cover). This was the opposite for leaf leachates, where the variance was explained mainly by the effect of environmental variables alone (PET, aridity, and dry period duration).

In samples from the temperate zone, variance of leachate quality was best explained for biofilms (27%) followed by leaves (13%) and sediments (6%) (Table 3). The same was found for the amounts of leached substances, where the explained variance for biofilms was due to the effect of environmental variables (PET and fraction of different land use types), and for leaves due to the effect of substrate characteristics (%C, %N). For sediments, the share of variance explained by the effect of substrate characteristics and the effect of environmental variables was approximately equal (VIP >1 for sediment texture classes, river width, altitude).

### 3.6 Estimated areal fluxes of nutrients and OM across IRES riverbeds

Area‐specific fluxes differed by two to four orders‐of‐magnitude among the sampled riverbeds, depending on the nutrient and OM species (Figure S1, Table 4). Fluxes of DOC and SRP differed by two orders‐of‐magnitude and ranged for DOC from 3 to 163 g/m^2^ riverbed surface (median: 15.2) and for SRP from 0.015 to 2.63 g/m^2^ (median: 0.12). Fluxes of N‐NH_4_^+ ^and phenolics spanned three orders‐of‐magnitude (N‐NH_4_^+^: 0.009–6.67 g/m^2^, median: 0.27; phenolics: 0.012–35 g/m^2^, median: 1.39). N‐NO_3_^− ^fluxes spanned the largest range, from 0.008 to 18.88 g/m^2^ (median: 0.59 g/m^2^). Overall, the released fluxes decreased in the following order: DOC > phenolics > N‐NO_3_^−^> N‐NH_4_^+^ > SRP.

**Table 4:** Comparison of the areal fluxes (g/m^2^) of the different nutrients and OM species across climate zones (for abbreviations see Table 1)

Parameter	Arid (*N* = 23)				Temperate (*N* = 105)				Tropical (*N* = 15)				Continental (*N* = 12)			
	Median	Mean ± SD	Min	Max	Median	Mean ± SD	Min	Max	Median	Mean ± SD	Min	Max	Median	Mean ± SD	Min	Max
DOC	9.40	11.00 ± 6.07	2.96	26.71	16.70	24.90 ± 29.82	3.00	162.67	15.90	14.99 ± 7.53	3.71	28.01	43.80	44.79 ± 21.15	15.04	82.58
N‐NH_4_^+^	0.22	0.29 ± 0.33	0.01	1.65	0.25	0.56 ± 0.92	0.01	6.67	0.33	0.42 ± 0.28	0.04	1.06	0.61	0.68 ± 0.23	0.43	1.24
N‐NO_3_^−^	0.41	0.65 ± 0.78	0.03	3.64	0.62	1.56 ± 2.76	0.01	18.87	0.78	1.39 ± 1.67	0.16	5.59	1.65	2.53 ± 2.92	0.03	11.31
SRP	0.07	0.12 ± 0.14	0.03	0.57	0.10	0.20 ± 0.34	0.02	2.63	0.11	0.15 ± 0.12	0.03	0.51	0.36	0.48 ± 0.37	0.15	1.48
Phenolics	1.10	1.57 ± 2.08	0.01	9.43	1.45	3.19 ± 4.95	0.012	35.00	1.11	1.90 ± 2.04	0.05	7.57	2.78	2.75 ± 1.19	0.37	4.58

Major contributions to the areal fluxes from riverbeds were made by sediments: 98 ± 7% (mean ± *SD*) for N‐NO_3_^−^, 97 ± 6% for N‐NH_4_^+^, 86 ± 19% for SRP, 85 ± 20% for DOC, and 56 ± 33% for phenolics. Leaves provided the second highest contribution to the total areal flux. In contrast to sediments and leaves, the relative contribution of biofilms to area‐specific flux rates was very low for all substances (in average: <0.1%), but slightly higher for N‐NO_3_^−^ (1.5 ± 7%) (values above 100% or lower than 0% reflect deviation and not the real data).

The highest fluxes were estimated from riverbeds in the continental zone (Table 4), whose areal flux of N‐NH_4_^+ ^and phenolics was three times higher than that of the arid zone, four times higher for N‐NO_3_^−^, and five times higher for SRP and DOC. For all nutrients and OM species, except phenolics (Kruskal–Wallis test, *χ* ^2^ = 4.68, *df* = 3, *p* = 0.2), the differences between continental and arid zones were statistically significant (Dunn's test, *p* < 0.001 for all pairwise comparisons). Compared to the continental zone, a lower flux was found for DOC in temperate and tropical zones (Kruskal–Wallis test, *χ* ^2^ =  24.8, *df* = 3, *p* = 0.003; Dunn's tests *p* = 0.001 and *p* = 0.005 respectively) and SRP (Kruskal–Wallis test, *χ* ^2^ = 20.02, *df* = 3, *p* < 0.001; Dunn's tests *p* = 0.001 and *p* = 0.004 respectively). The flux of N‐NH_4_^+ ^was lower in the temperate zone than in the continental zone (Kruskal–Wallis test, *χ* ^2^ = 16.5, *df* = 3, *p* < 0.001; Dunn's test *p* = 0.006).

## 4 DISCUSSION

### 4.1 Rewetting events in IRES in the context of global biogeochemical cycles

Our globally comparable assessment of nutrient and DOM leaching in rewetted IRES shows that the quantity and quality of leached nutrients and DOM are substrate‐ and climate‐specific, with the highest amounts leached in continental climate and with sediments contributing most to the total areal flux from dry river beds. These data provide a basis on which to develop models of biogeochemical cycling in river networks including IRES.

According to our first hypothesis, we found a high variability in the amount of leached substances and the quality of leachates from organic, but also from inorganic substrates, mainly as a consequence of inherent substrate properties and their modification during the drying period. Leaching from organic materials (leaves and biofilms) was relatively enriched in P vs N in contrast to sediments. Due to their higher mass within the riverbeds, sediments were the main contributors to the areal fluxes. Sediments leached high amounts of N‐NO_3_^‐^, the accumulation of which in dry riverbeds is promoted by aerobic conditions (Amalfitano et al., 2008; Arce et al., 2014; Borken & Matzner, 2009; Merbt et al., 2016). Considering quality of leached DOM, we found that depending on the proportion of each substrate within the riverbed, different ecosystem processes can be affected. For example, leachates from biofilms with a high proportion of biopolymers may play a key role as sources of bioavailable DOM in IRES and are more likely to be retained within the riverbed upon rewetting (Romani, Vazquez, & Butturini, 2006; von Schiller et al., 2015). A high proportion of LMWS leached from leaves suggests that such leachates can trigger ecosystem processes in downstream surface waters and groundwaters, as molecules of this size fraction can easily be transported through the hyporheic zone with limited immobilization (Romani et al., 2006). DOM leached from sediments was mainly of microbial origin, suggesting its high potential bioavailability (Marxsen, Zoppini, & Wilczek, 2010; Schimel et al., 2007). Overall, we suggest that rewetting of sediments is key for understanding biogeochemical cycles in fluvial networks with IRES, and that leaves and biofilms can introduce regional variabilities in the global scale patterns depending on the accumulated amount of these substrates in the channel during the dry phase. Indeed, accumulation of plant litter on the dry riverbed ranges from 0 to 963 g/m^2^ depending on aridity, river width, catchment area, riparian cover, and drying duration (Datry et al., 2018 and Table S1). In our study, accumulations of biofilms were very common in the temperate zone and ranged from 0.3 to 327 g/m^2 ^(Table S1).

We also found differences in the amounts of leached substances among climate zones, in accordance with our second hypothesis, but only for sediments. Initially, we expected cross‐climate differences to be more pronounced for leaves due to climatic effects on vegetation composition and leaf litter quality (e.g. Aerts, 1997; Boyero et al., 2017), rather than for sediments whose composition is controlled mainly by geology and geomorphology. The absence of significant differences among climate zones for leaves could be explained by the considerable variability we observed among leaf material collected within climate zones, both in terms of species composition and drying history. Although we did not assess the site‐specific composition of riparian vegetation, previous studies indicated that up to 40% of variation in leaf traits at a given site can be explained by small‐scale spatial and temporal environmental heterogenity in environmental factors such as hydrology and disturbance regime (Cornwell et al., 2008).

High concentrations leached in the continental climate zone suggest that nutrient loads to freshwaters will increase with the projected increase in the extent of IRES in such regions. In the arid zone where terrestrial primary production is severely constrained by water availability (Austin et al., 2004), rewetting events are expected to stimulate stream ecosystem productivity not only due to water availability, but also because the potential bioavailability of leachates is particularly high in this climate zone. However, despite a high potential bioavailability of DOM, leachates from the arid zone were characterized by low amounts of nutrients, probably resulting from leaf traits that reflect adaptation to dry conditions (Cornwell et al., 2008).

Comparison of fluxes from 1 m^2^ of IRES within the 4 hr duration of the experiment with the annual flux from 1 m^2 ^of watersheds (Table S5) showed that rewetting events in IRES represent a significant pulse of dissolved substances in ecosystems, including some estimates exceeding known annual fluxes from watersheds with perennial rivers (although differences in the size of watersheds and stream area of IRES should be accounted). While there can be some confounding factors between laboratory conditions and those that occur in a natural setting (i.e. intensity and duration of rewetting events, ambient temperature, increased leaching caused by oven‐drying (Gessner & Schwoerbel, 1989), presence of terrestrial plants in dry riverbeds (Gómez, Arce, Sánchez, & del Mar Sánchez‐Montoya, 2012)), the results of our experiment across various climate regions indicate that rewetting of IRES produces a pulsed release of dissolved substances. Decomposition of substrates accumulated in IRES, and thus carbon turnover, are affected by drying‐rewetting cycles (Fierer & Schimel, 2002). Given the predicted increase in the duration of droughts, the exacerbation of extreme low‐flow conditions, and the intensity of storm events (De Girolamo, Bouraoui, Buffagni, Pappagallo, & Lo Porto, 2017; Huntington, 2006; IPCC, 2014), the results of this study emphasize the need to integrate IRES in global carbon cycles and budgets, from which they are currently excluded (Raymond et al., 2013; although see Datry et al., 2018).

### 4.2 Environmental variables correlated with release of nutrients and OM

Environmental variables that are prone to be affected by climate change (namely PET, aridity, dry period duration, land‐use) correlated with amounts and quality of leachates, particularly for sediments. For leaves, these correlations were less pronounced, suggesting that leaching may be affected by substrate characteristics other than those examined here. Characteristics such as toughness and content of secondary metabolites in substrates could have affected leaching through the effect on their mass loss during the dry phase and simulated rewetting, and on activity of microbial community in leachates (e.g. Pérez‐Harguindeguy et al., 2000; Ristock et al., 2017). Latitude, although not considered in the study, may also be responsible for the unexplained variance given that litter quality generally increases with latitude (Boyero et al., 2017).

The amounts of leached substances from both leaves and sediments were correlated with PET. This variable is expected to be intensified in the future (Milly & Dunne, 2016) and will most likely lead to fluctuations in moisture conditions in dry riverbeds. Low moisture level reduces litter decomposition and C consumption, thereby promoting the release of DOM upon rewetting (Abril et al., 2016; Aerts, 1997; Bruder et al., 2011; Gessner, 1991) and hence increasing the probability of negative consequences for stream ecosystems such as blackwater events leading to hypoxia (Hladyz et al., 2011).

Differences among climate zones in terms of correlations of environmental variables with amounts of leached substances indicate that climate change can have different effects on IRES in different geographical regions. For example, in the arid zone, where IRES are usually characterized by open canopy (Steward, Schiller, Tockner, Marshall, & Bunn, 2012), aridity and percentage of riparian vegetation best explained the variance in sediment leachates. Inputs of riparian vegetation litter onto the dry riverbeds and its subsequent decomposition, can represent an additional input of nutrients to sediments in the arid zone areas (Abril et al., 2016), where soils generally contain less carbon and nitrogen compared to the continental zone (Table S1 and Delgado‐Baquerizo et al., 2013). Changes in land‐use (particularly, in the percentage of pasture cover at the global scale as well as within individual climate zones except continental) were correlated with the amount of leached substances from leaves, potentially through modifying the composition of plant material accumulated in beds of IRES. This suggests that modification of land use in the catchments with IRES can also affect their contribution to nutrient load due to changes in the composition of CPOM accumulating in dry riverbeds.

Although dry period duration is an important factor affecting the amounts and quality of litter accumulations in IRES (del Campo & Gómez, 2016; von Schiller et al.,^2017^), we found its influence on the variance in leachates only in continental and tropical zones. This indicates that during the dry phase materials with different drying history (as affected by different climates) and potential to leach nutrients and OM can accumulate in IRES. This also suggests that dry period duration cannot invariably be used as a master proxy to assess potential impacts of nutrient loading from IRES upon rewetting. Under field conditions, other factors such as severity and timing of a rewetting event as well as presence/absence of plant material growing in dry channels can affect nutrient fluxes from riverbeds, and the fate of nutrients in ecosystems, as well as potential ecosystem impacts (e.g. eutrophication, mass mortality of aquatic organisms) in downstrean receiving waters and groundwater (Baldwin & Mitchell, 2000; Bernal et al., 2013; Cavanaugh, Richardson, Strauss, & Bartsch, 2006; Hladyz et al., 2011; Ocampo, Oldham, Sivapalan, & Turner, 2006). Substrate moisture content and variability in associated microbial communities can potentially be responsible for the unexplained part of the variance in the leachates, due to their effect on decomposition rates of accumulated CPOM, nutrient processing in sediments, release of DOM upon rewetting, and its modification by microbial communities (Abril et al., 2016; Arce et al., 2015; Dieter, Frindte, Krüger, & Wurzbacher, 2013; McIntyre, Adams, Ford, & Grierson, 2009; Meisner, Leizeaga, Rousk, & Bååth, 2017).

### 4.3 Implications for freshwater ecosystems and future research

We identified IRES to function as pulsed biogeochemical reactors (sensu Larned et al., 2010) at a global scale even though the experiments were conducted under laboratory conditions and magnitudes of leached substances may differ in the natural environment. Our data serve also as a basis for further upscaling and modeling of the processes observed in the laboratory to address ecological implications of rewetting events at catchment scales. Potential implications for the functioning of rivers could be determined by the effect of leached substances on the degree of nutrient limitation of microorganisms downstream, and therefore community composition (Demi, Benstead, Rosemond, & Maerz, 2018) as well as on the fate of refractory substances and intensification of their decomposition through the so‐called “priming effect” (Guenet, Danger, Abbadie, & Lacroix, 2010). The results of our study support the recent call for developing effective strategies for the management of IRES to avoid negative consequences for downstream ecosystems caused by excessive nutrient and OM load.

## References

[gcb14537-bib-0001] Abril, M. , Muñoz, I. , & Menéndez, M. (2016). Heterogeneity in leaf litter decomposition in a temporary Mediterranean stream during flow fragmentation. Science of the Total Environment, 553, 330–339. 10.1016/j.scitotenv.2016.02.082 26930306

[gcb14537-bib-0002] Acuña, V. , Datry, T. , Marshall, J. , Barceló, D. , Dahm, C. N. , Ginebreda, A. , … Palmer, M. A. (2014). Why should we care about temporary waterways? Science, 343, 1080–1081. 24604183 10.1126/science.1246666

[gcb14537-bib-0003] Aerts, R. (1997). Climate, leaf litter chemistry and leaf litter decomposition in terrestrial ecosystems: A triangular relationship. Oikos, 79, 439–449. 10.2307/3546886

[gcb14537-bib-0004] Allan, J. D. (2004). Landscapes and riverscapes: The influence of land use on stream ecosystems. Annual Review of Ecology, Evolution and Systematics, 35, 257–284. 10.1146/annurev.ecolsys.35.120202.110122

[gcb14537-bib-0005] Amalfitano, S. , Fazi, S. , Zoppini, A. M. , Caracciolo, A. B. , Grenni, P. , & Puddu, A. (2008). Responses of benthic bacteria to experimental drying in sediments from Mediterranean temporary rivers. Microbial Ecology, 55, 270–279. 10.1007/s00248-007-9274-6 17603744

[gcb14537-bib-0006] Arce, M. I. , Sánchez‐Montoya, M. M. , & Gómez, R. (2015). Nitrogen processing following experimental sediment rewetting in isolated pools in an agricultural stream of a semiarid region. Ecological Engineering, 77, 233–241. 10.1016/j.ecoleng.2015.01.035

[gcb14537-bib-0007] Arce, M. , Sánchez‐Montoya, M. M. , Vidal‐Abarca, M. R. , Suárez, M. L. , & Gómez, R. (2014). Implications of flow intermittency on sediment nitrogen availability and processing rates in a Mediterranean headwater stream. Aquatic Science, 76, 173–186. 10.1007/s00027-013-0327-2

[gcb14537-bib-0008] Arnell, N. W. , & Gosling, S. N. (2013). The impacts of climate change on river flow regimes at the global scale. Journal of Hydrology, 486, 351–364. 10.1016/j.jhydrol.2013.02.010

[gcb14537-bib-0009] Austin, A. T. , Yahdjian, L. , Stark, J. M. , Belnap, J. , Porporato, A. , Norton, U. , … Schaeffer, S. M. (2004). Water pulses and biogeochemical cycles in arid and semiarid ecosystems. Oecologia, 141, 221–235. 10.1007/s00442-004-1519-1 14986096

[gcb14537-bib-0010] Baldwin, D. S. , & Mitchell, A. M. (2000). The effects of drying and re‐flooding on the sediment and soil nutrient dynamics of lowland river‐floodplain systems: A synthesis. Regulated Rivers: Research and Management, 16, 457–467. 10.1002/1099-1646(200009/10)16:5<457:AID-RRR597>3.0.CO;2-B

[gcb14537-bib-0011] Bärlocher, F. (2005). Leaching In Graça M. A. S., Bärlocher F., & Gessner M. O. (Eds.), Methods to study litter decomposition: A practical guide (pp. 33–36). The Netherlands: Springer.

[gcb14537-bib-0012] Bernal, S. , von Schiller, D. , Sabater, F. , & Martí, E. (2013). Hydrological extremes modulate nutrient dynamics in mediterranean climate streams across different spatial scales. Hydrobiologia, 719, 31–42. 10.1007/s10750-012-1246-2

[gcb14537-bib-0013] Bernhardt, E. S. , Heffernan, J. B. , Grimm, N. B. , Stanley, E. H. , Harvey, J. W. , Arroita, M. , … Yackulic, C. B. (2018). The metabolic regimes of flowing waters. Limnology and Oceanography, 63, 99–118. 10.1002/lno.10726

[gcb14537-bib-0014] Boix‐Fayos, C. , Nadeu, E. , Quiñonero, J. M. , Martínez‐Mena, M. , Almagro, M. , & de Vente, J. (2015). Sediment flow paths and associated organic carbon dynamics across a Mediterranean catchment. Hydrology and Earth System Sciences, 19, 1209–1223. 10.5194/hess-19-1209-2015

[gcb14537-bib-0015] Borcard, D. , Legendre, P. , & Drapeau, P. (1992). Partialling out the spatial component of ecological variation. Ecology, 73, 1045–1055. 10.2307/1940179

[gcb14537-bib-0016] Borken, W. , & Matzner, E. (2009). Reappraisal of drying and wetting effects on C and N mineralization and fluxes in soils. Global Change Biology, 15, 808–824. 10.1111/j.1365-2486.2008.01681.x

[gcb14537-bib-0017] Boulton, A. J. , & Boon, P. I. (1991). A review of methodology used to measure leaf litter decomposition in lotic environments – Time to turn over an old leaf. Australian Journal of Marine and Freshwater Research, 42, 1591–43. 10.1071/MF9910001

[gcb14537-bib-0018] Boyero, L. , Graça, M. , Tonin, A. M. , Pérez, J. , Swafford, A. J. , Ferreira, V. , … Pearson, R. G. , (2017). Riparian plant litter quality increases with latitude. Scientific Reports, 7(1), 10562 10.1038/s41598-017-10640-3 28874830 PMC5585321

[gcb14537-bib-0019] Bruder, A. , Chauvet, E. , & Gessner, M. O. (2011). Litter diversity, fungal decomposers and litter decomposition under simulated stream intermittency. Functional Ecology, 25, 1269–1277. 10.1111/j.1365-2435.2011.01903.x

[gcb14537-bib-0020] Bunn, S. E. , Thoms, M. C. , Hamilton, S. K. , & Capon, S. J. (2006). Flow variability in dryland rivers: Boom, bust and the bits in between. River Research and Application, 22, 179–186. 10.1002/rra.904

[gcb14537-bib-0021] Catalan, N. , Obrador, B. , Alomar, C. , & Pretus, J. L. (2013). Seasonality and landscape factors drive dissolved organic matter properties in Mediterranean ephemeral washes. Biogeochemistry, 112, 261–274. 10.1007/s10533-012-9723-2

[gcb14537-bib-0022] Cavanaugh, J. C. , Richardson, W. B. , Strauss, E. A. , & Bartsch, L. A. (2006). Nitrogen dynamics in sediment during water level manipulation on the upper Missisippi River. River Research and Applications, 22, 651–666.

[gcb14537-bib-0023] Chiu, M. C. , Leigh, C. , Mazor, R. , Cid, N. , & Resh, V. (2017). Anthropogenic threats to intermittent rivers and ephemeral streams In Datry T., Bonada N., & Boulton A. (Eds.), Intermittent rivers and ephemeral streams: Ecology and Management (pp. 433–454). London, UK: Academic Press.

[gcb14537-bib-0024] Conley, D. J. , Paerl, H. W. , Howarth, R. W. , Boesch, D. F. , Seitzinger, S. P. , Havens, K. E. , … Likens, G. E. (2009). Controlling eutrophication: Nitrogen and phosphorus. Science, 323, 1014–1015. 19229022 10.1126/science.1167755

[gcb14537-bib-0025] Cornwell, W. K. , Cornelissen, J. H. C. , Amatangelo, K. , Dorrepaal, E. , Eviner, V. T. . … Westoby, M. (2008). Plant species traits are the predominant control on litter decomposition rates within biomes worldwide. Ecology Letters, 11, 1065–1071. 10.1111/j.1461-0248.2008.01219.x 18627410

[gcb14537-bib-0026] Corti, R. , & Datry, T. (2012). Invertebrates and sestonic matter in an advancing wetted front travelling down a dry river bed (Albarine, France). Freshwater Science, 31, 1187–1201. 10.1899/12-017.1

[gcb14537-bib-0028] Datry, T. , Bonada, N. , & Boulton, A. J. (2017). General introduction In Datry T., Bonada N., & Boulton A. (Eds.), Intermittent rivers and ephemeral streams (pp. 1591–20). London, UK: Academic Press.

[gcb14537-bib-0029] Datry, T. , Corti, R. , Foulquier, A. , von Schiller, D. , & Tockner, T. (2016). One for all, all for one: A global river research network. EOS: Earth & Space Science News, 97, 13–15.

[gcb14537-bib-0027] Datry, T. , Foulquier, A. , Corti, R. , von Schiller, D. , Tockner, K. , Mendoza-Lera, C. , … Zoppini, A. (2018). A global analysis of terrestrial plant litter dynamics in non-perennial waterways. Nature Geoscience, 11, 497–503. 10.1038/s41561-018-0134-4

[gcb14537-bib-0030] Datry, T. , Larned, S. T. , & Tockner, K. (2014). Intermittent rivers: A challenge for freshwater ecology. BioScience, 64, 229–235. 10.1093/biosci/bit027

[gcb14537-bib-0031] De Girolamo, A. M. , Bouraoui, F. , Buffagni, A. , Pappagallo, G. , & Lo Porto, A. (2017). Hydrology under climate change in a temporary river system: Potential impact on water balance and flow regime. River Research and Applications, 33, 1219–1232.

[gcb14537-bib-0032] del Campo, R. , & Gómez, R. (2016). Exposure of wood in floodplains affects its chemical quality and its subsequent breakdown in streams. Science of the Total Environment, 543, 652–661. 10.1016/j.scitotenv.2015.11.050 26613519

[gcb14537-bib-0033] Delgado‐Baquerizo, M. , Maestre, F. T. , Gallardo, A. , Bowker, M. A. , Wallenstein, M. D. , Quero, J. L. , … Zaady, E. (2013). Decoupling of soil nutrient cycles as a function of aridity in global drylands. Nature, 502, 672–676. 10.1038/nature12670 24172979

[gcb14537-bib-0034] Demi, L. M. , Benstead, J. P. , Rosemond, A. D. , & Maerz, J. C. (2018). Litter P content drives consumer production in detritus based streams spanning an experimental N: P gradient. Ecology, 99, 347–359. 10.1002/ecy.2118 29266195

[gcb14537-bib-0035] Dieter, D. , Frindte, K. , Krüger, A. , & Wurzbacher, C. (2013). Preconditioning of leaves by solar radiation and anoxia affects microbial colonisation and rate of leaf mass loss in an intermittent stream. Freshwater Biology, 58, 1918–1931. 10.1111/fwb.12180

[gcb14537-bib-0036] Dieter, D. , von Schiller, D. , Garcia‐Roger, E. , Sanchez‐Montoya, M. M. , Gomez, R. , Mora‐Gomez, J. , … Tockner, K. (2011). Preconditioning effects of intermittent stream flow on leaf litter decomposition. Aquatic Sciences, 73, 599–609. 10.1007/s00027-011-0231-6

[gcb14537-bib-0037] Dunn, O. J. (1964). Multiple comparisons using rank sums. Technometrics, 6, 241–252. 10.1080/00401706.1964.10490181

[gcb14537-bib-0038] Elser, J. J. , Bracken, M. E. S. , Cleland, E. E. , Gruner, D. S. , Harpole, W. S. , Hillebrand, H. , … Smith, J. E. (2007). Global analysis of nitrogen and phosphorus limitation of primary producers in freshwater, marine and terrestrial ecosystems. Ecology Letters, 10, 1135–1142. 17922835 10.1111/j.1461-0248.2007.01113.x

[gcb14537-bib-0039] Eriksson, L. , Johansson, E. , Kettaneh‐Wold, N. , & Wold, S. (2006). Multi‐ and megavariate data analysis: Principles and applications. Umea, Sweden: Umetrics AB.

[gcb14537-bib-0040] Fellman, J. B. , Hood, E. , & Spencer, R. G. M. (2010). Fluorescence spectroscopy opens new windows into dissolved organic matter dynamics in freshwater ecosystems: A review. Limnology and Oceanography, 55, 2452–2462. 10.4319/lo.2010.55.6.2452

[gcb14537-bib-0041] Fellman, J. B. , Petrone, K. C. , & Grierson, P. F. (2013). Leaf litter age, chemical quality, and photodegradation control the fate of leachate dissolved organic matter in a dryland river. Journal of Arid Environments, 89, 30–37. 10.1016/j.jaridenv.2012.10.011

[gcb14537-bib-0042] Fierer, N. , & Schimel, J. P. (2002). Effects of drying‐rewetting frequency on soil carbon and nitrogen transformations. Soil Biology & Biochemistry, 34, 777–787. 10.1016/S0038-0717(02)00007-X

[gcb14537-bib-0043] Gessner, M. O. (1991). Differences in processing dynamics of fresh and dried leaf litter in a stream ecosystem. Freshwater Biology, 26, 387–398. 10.1111/j.1365-2427.1991.tb01406.x

[gcb14537-bib-0044] Gessner, M. O. , & Schwoerbel, J. (1989). Leaching kinetics of fresh leaf‐litter with implications for the current concept of leaf‐processing in streams. Archiv Für Hydrobiologie, 115, 81–90.

[gcb14537-bib-0045] Gómez, R. , Arce, M. I. , Sánchez, J. J. , & del Mar Sánchez‐Montoya, M. (2012). The effects of drying on sediment nitrogen content in a Mediterranean intermittent stream: A microcosms study. Hydrobiologia, 679, 43–59. 10.1007/s10750-011-0854-6

[gcb14537-bib-0046] Guenet, B. , Danger, M. , Abbadie, L. , & Lacroix, G. (2010). Priming effect: Bridging the gap between terrestrial and aquatic ecology. Ecology, 91, 2850–2861. 10.1890/09-1968.1 21058546

[gcb14537-bib-0047] Hansen, A. M. , Kraus, T. E. C. , Pellerin, B. A. , Fleck, J. A. , Downing, B. D. , & Bergamaschi, B. A. (2016). Optical properties of dissolved organic matter (DOM): Effects of biological and photolytic degradation. Limnology and Oceanography, 61, 1015–1032. 10.1002/lno.10270

[gcb14537-bib-0048] Harris, C. W. , Silvester, E. , Rees, G. N. , Pengelly, J. , & Puskar, L. (2016). Proteins are a major component of dissolved organic nitrogen (DON) leached from terrestrially aged Eucalyptus camaldulensis leaves. Environmental Chemistry, 13, 877–887. 10.1071/EN16005

[gcb14537-bib-0049] Hillel, D. (1980). Fundamentals of soils physics. New York, NY: Academic Press.

[gcb14537-bib-0050] Hladyz, S. , Watkins, S. C. , Whitworth, K. L. , & Baldwin, D. S. (2011). Flows and hypoxic blackwater events in managed ephemeral river channels. Journal of Hydrology, 401, 117–125. 10.1016/j.jhydrol.2011.02.014

[gcb14537-bib-0051] Huntington, T. G. (2006). Evidence for intensification of the global water cycle: Review and synthesis. Journal of Hydrology, 319, 83–95. 10.1016/j.jhydrol.2005.07.003

[gcb14537-bib-0052] IPCC . (2014). Summary for policymakers In Edenhofer O, Pichs-Madruga R, Sokona Y, Farahani E., Kadner S., Seyboth K, … Minx J. C. (Eds.), Climate change 2014: Mitigation of climate change. Contribution of working group III to the fifth assessment report of the intergovernmental panel on climate change (pp. 1591–32). Cambridge, UK: Cambridge University Press.

[gcb14537-bib-0053] Jacobson, P. J. , & Jacobson, K. M. (2013). Hydrologic controls of physical and ecological processes in Namib Desert ephemeral rivers: Implications for conservation and management. Journal of Arid Environments, 93, 80–93. 10.1016/j.jaridenv.2012.01.010

[gcb14537-bib-0054] Kuiters, A. T. , & Sarink, H. M. (1986). Leaching of phenolic compounds from leaf and needle litter of several deciduous and coniferous trees. Soil Biology & Biochemistry, 18, 475–480. 10.1016/0038-0717(86)90003-9

[gcb14537-bib-0055] Larned, S. T. , Datry, T. , Arscott, D. B. , & Tockner, K. (2010). Emerging concepts in temporary‐river ecology. Freshwater Biology, 55, 717–738. 10.1111/j.1365-2427.2009.02322.x

[gcb14537-bib-0056] Leigh, C. , Boulton, A. J. , Courtwright, J. L. , Fritz, K. , May, C. L. , Walker, R. H. , & Datry, T. (2016). Ecological research and management of intermittent rivers: An historical review and future directions. Freshwater Biology, 61, 1181–1199. 10.1111/fwb.12646

[gcb14537-bib-0057] Marxsen, J. , Zoppini, A. , & Wilczek, S. (2010). Microbial communities in streambed sediments recovering from desiccation. FEMS Microbiology Ecology, 71, 374–386. 10.1111/j.1574-6941.2009.00819.x 20041952

[gcb14537-bib-0058] McIntyre, R. , Adams, M. A. , Ford, D. J. , & Grierson, P. F. (2009). Rewetting and litter addition influence mineralization and microbial communities in soils from a semiarid intermittent stream. Soil Biology & Biochemistry, 41, 92–101.

[gcb14537-bib-0059] McKnight, D. M. , Boyer, E. W. , Westerhoff, P. K. , Doran, P. T. , Kulbe, T. , & Andersen, D. T. (2001). Spectrofluorometric characterization of dissolved organic matter for indication of precursor organic material and aromaticity. Limnology and Oceanography, 46, 38–48. 10.4319/lo.2001.46.1.0038

[gcb14537-bib-0060] McNamara, C. J. , & Leff, L. G. (2004). Bacterial community composition in biofilms on leaves in a northeastern Ohio stream. Journal of the North American Benthological Society, 23, 677–685. 10.1899/0887-3593(2004)023<0677:BCCIBO>2.0.CO;2

[gcb14537-bib-0061] Meisner, A. , Leizeaga, A. , Rousk, J. , & Bååth, E. (2017). Partial drying accelerates bacterial growth recovery to rewetting. Soil Biology & Biochemistry, 112, 269–276. 10.1016/j.soilbio.2017.05.016

[gcb14537-bib-0062] Merbt, S. N. , Proia, L. , Prosser, J. I. , Casamayor, E. O. , & von Schiller, D. (2016). Stream drying drives microbial ammonia oxidation and first flush nitrate export. Ecology, 97, 2192–2198. 10.1002/ecy.1486 27859084

[gcb14537-bib-0063] Milly, P. C. D. , & Dunne, K. A. (2016). Potential evapotranspiration and continental drying. Nature Climate Change, 6, 946–949.

[gcb14537-bib-0064] Milly, P. C. D. , Dunne, K. A. , & Vecchia, A. V. (2005). Global pattern of trends in streamflow and water availability in a changing climate. Nature, 438, 347–350. 10.1038/nature04312 16292308

[gcb14537-bib-0065] Nykvist, N. (1963). Leaching and decomposition of water‐soluble organic substances from different types of leaf and needle litter. Studia Forestalia Suecica, 3, 1591–31.

[gcb14537-bib-0066] Obermann, M. , Froebrich, J. , Perrin, J.‐L. , & Tournoud, M.‐J. (2007). Impact of significant floods on the annual load in an agricultural catchment in the Mediterranean. Journal of Hydrology, 334, 99–108. 10.1016/j.jhydrol.2006.09.029

[gcb14537-bib-0067] Ocampo, C. J. , Oldham, C. E. , Sivapalan, M. , & Turner, J. V. (2006). Hydrological versus biogeochemical controls on catchment nitrate export: A test of the flushing mechanism. Hydrological Processes, 20, 4269–4286. 10.1002/hyp.6311

[gcb14537-bib-0068] Ohno, T. (2002). Fluorescence inner‐filtering correction for determining the humification index of dissolved organic matter. Environmental Science & Technology, 36, 742–746. 10.1021/es0155276 11878392

[gcb14537-bib-0069] Ostojić, A. , Rosado, J. , Miliša, M. , Morais, M. , & Tockner, K. (2013). Release of nutrients and organic matter from river floodplain habitats: Simulating seasonal inundation dynamics. Wetlands, 33, 847–859. 10.1007/s13157-013-0442-9

[gcb14537-bib-0070] Parlanti, E. , Worz, K. , Geoffroy, L. , & Lamotte, M. (2000). Dissolved organic matter fluorescence spectroscopy as a tool to estimate biological activity in a coastal zone submitted to anthropogenic inputs. Organic Geochemistry, 31, 1765–1781. 10.1016/S0146-6380(00)00124-8

[gcb14537-bib-0071] Pekel, J.‐F. , Cottam, A. , Gorelick, N. , & Belward, A. S. (2016). High‐resolution mapping of global surface water and its long‐term changes. Nature, 540, 418–422. 10.1038/nature20584 27926733

[gcb14537-bib-0072] Pérez‐Harguindeguy, N. , Díaz, S. , Cornelissen, J. H. C. , Vendramini, F. , Cabido, M. , & Castellanos, A. (2000). Chemistry and toughness predict leaf litter decomposition rates over a wide spectrum of functional types and taxa in central Argentina. Plant and Soil, 218, 21–30.

[gcb14537-bib-0073] R Core Team . (2017). R: A language and environment for statistical computing. Vienna, Austria: R Foundation for Statistical Computing.

[gcb14537-bib-0074] Raymond, P. A. , Hartmann, J. , Lauerwald, R. , Sobek, S. , McDonald, C. , Hoover, M. , … Guth, P. (2013). Global carbon dioxide emissions from inland waters. Nature, 503, 355–359. 24256802 10.1038/nature12760

[gcb14537-bib-0075] Ristok, C. , Leppert, K. N. , Franke, K. , Scherer‐Lorenzen, M. , Niklaus, P. A. , Wessjohann, L. A. , & Bruelheide, H. (2017). Leaf litter diversity positively affects the decomposition of plant polyphenols. Plant and Soil, 419, 305–317. 10.1007/s11104-017-3340-8

[gcb14537-bib-0076] Romani, A. M. , Vazquez, E. , & Butturini, A. (2006). Microbial availability and size fractionation of dissolved organic carbon after drought in an intermittent stream: Biogeochemical link across the stream‐riparian interface. Microbial Ecology, 52, 501–512. 10.1007/s00248-006-9112-2 16897299

[gcb14537-bib-0077] Romani, A. M. , Chauvet, E. , Febria, C. , Mora-Gomez, J. , Risse-Buhl, U. , Timoner, X. , & Zeglin, L. (2017). The biota of intermittent rivers and ephemeral streams: Prokaryotes, fungi, and protozoans In Datry T., Bonada N., & Boulton A. (Eds.), Intermittent rivers and ephemeral streams (pp. 161–188). London, UK: Academic Press.

[gcb14537-bib-0078] Rosado, J. , Morais, M. , & Tockner, K. (2015). Mass dispersal of terrestrial organisms during first flush events in a temporary stream. River Research and Application, 31, 912–917.

[gcb14537-bib-0079] Sabater, S. , Timoner, X. , Borrego, C. , & Acuña, V. (2016). Stream biofilm responses to flow intermittency: From cells to ecosystems. Frontiers in Environmental Science, 4, 1591–14. 10.3389/fenvs.2016.00014

[gcb14537-bib-0080] Schimel, J. , Balser, T. C. , & Wallenstein, M. (2007). Microbial stress‐response physiology and its implications for ecosystem function. Ecology, 88, 1386–1394. 10.1890/06-0219 17601131

[gcb14537-bib-0081] Skoulikidis, N. , & Amaxidis, Y. (2009). Origin and dynamics of dissolved and particulate nutrients in a minimally disturbed Mediterranean river with intermittent flow. Journal of Hydrology, 373, 218–229. 10.1016/j.jhydrol.2009.04.032

[gcb14537-bib-0082] Skoulikidis, N. , Vardakas, L. , Amaxidis, Y. , & Michalopoulos, P. (2017). Biogeochemical processes controlling aquatic quality during drying and rewetting events in a Mediterranean non‐perennial river reach. Science of the Total Environment, 575, 378–389. 27750134 10.1016/j.scitotenv.2016.10.015

[gcb14537-bib-0083] Skoulikidis, N. T. , Sabater, S. , Datry, T. , Morais, M. M. , Buffagni, A. , Dörflinger, G. , … Tockner, K. (2017). Non‐perennial Mediterranean rivers in Europe: Status, pressures, and challenges for research and management. Science of the Total Environment, 577, 1591–18. 10.1016/j.scitotenv.2016.10.147 27810301

[gcb14537-bib-0084] Soria, M. , Leigh, C. , Datry, T. , Bini, L. M. , & Bonada, N. (2017). Biodiversity in perennial and intermittent rivers: A meta‐analysis. Oikos, 126, 1078–1089. 10.1111/oik.04118

[gcb14537-bib-0085] Steward, A. L. , von Schiller, D. , Tockner, K. , Marshall, J. C. , & Bunn, S. E. (2012). When the river runs dry: Human and ecological values of dry riverbeds. Frontiers in Ecology and the Environment, 10, 202–209. 10.1890/110136

[gcb14537-bib-0086] Taylor, B. R. , & Bärlocher, F. (1996). Variable effects of air‐drying on leaching losses from tree leaf litter. Hydrobiologia, 325, 173–182. 10.1007/BF00014982

[gcb14537-bib-0087] Tonkin, J. D. , Merritt, D. M. , Olden, J. D. , Reynolds, L. V. , & Lytle, D. A. (2018). Flow regime alteration degrades ecological networks in riparian ecosystems. Nature Ecology and Evolution, 2, 86–93. 29180707 10.1038/s41559-017-0379-0

[gcb14537-bib-0088] Tzoraki, O. , Nikolaidis, N. P. , Amaxidis, Y. , & Skoulikidis, N. T. (2007). Instream biogeochemical processes of a temporary river. Environmental Science and Technology, 41, 1225–1231. 10.1021/es062193h 17593723

[gcb14537-bib-0089] von Schiller, D. , Bernal, S. , Dahm, C. N. , & Martí, E. (2017). Nutrient and organic matter dynamics in intermittent rivers In Datry T., Bonada N., & Boulton A. (Eds.), Intermittent rivers and ephemeral streams (pp. 135–160). London, UK: Academic Press.

[gcb14537-bib-0090] von Schiller, D. , Acuña, V. , Graeber, D. , Martí, E. , Ribot, M. , Sabater, S. , … Tockner, K. (2011). Contraction, fragmentation and expansion dynamics determine nutrient availability in a Mediterranean forest stream. Aquatic Sciences, 73, 485–497. 10.1007/s00027-011-0195-6

[gcb14537-bib-0091] von Schiller, D. , Graeber, D. , Ribot, M. , Timoner, X. , Acuña, V. , Marti, E. , … Tockner, K. (2015). Hydrological transitions drive dissolved organic matter quantity and composition in a temporary Mediterranean stream. Biogeochemistry, 123, 429–446. 10.1007/s10533-015-0077-4

[gcb14537-bib-0092] Weishaar, J. L. , Aiken, G. R. , Bergamaschi, B. A. , Fram, M. S. , Fujii, R. , & Mopper, K. (2003). Evaluation of specific ultraviolet absorbance as an indicator of the chemical composition of dissolved organic matter. Environmental Science and Technology, 37, 4702–4708. 14594381 10.1021/es030360x

[gcb14537-bib-0093] Whitworth, K. L. , Baldwin, D. S. , & Kerr, J. L. (2012). Drought, floods and water quality: Drivers of a severe hypoxic blackwater event in a major river system (the southern Murray‐Darling Basin, Australia). Journal of Hydrology, 450–451, 190–198. 10.1016/j.jhydrol.2012.04.057

[gcb14537-bib-0094] Wilson, H. F. , & Xenopoulos, M. A. (2008). Ecosystem and seasonal control of stream dissolved organic carbon along a gradient of land use. Ecosystems, 11, 555–568. 10.1007/s10021-008-9142-3

[gcb14537-bib-0095] Wold, S. , Sjöström, M. , & Eriksson, L. (2001). PLS‐regression: A basic tool of chemometrics. Chemometrics and Intelligent Laboratory Systems, 58, 109–130. 10.1016/S0169-7439(01)00155-1

[gcb14537-bib-0096] XLSTAT . (2017). Data analysis and statistical solution for microsoft excel. Paris, France: Addinsoft.

[gcb14537-bib-0097] Zsolnay, A. , Baigar, E. , Jimenez, M. , Steinweg, B. , & Saccomandi, F. (1999). Differentiating with fluorescence spectroscopy the sources of dissolved organic matter in soils subjected to drying. Chemosphere, 38, 45–50. 10.1016/S0045-6535(98)00166-0 10903090

[gcb14537-bib-0098] Zuur, A. F. , Ieno, E. N. , & Elphick, C. S. (2010). A protocol for data exploration to avoid common statistical problems. Methods in Ecology and Evolution, 1, 3–14. 10.1111/j.2041-210X.2009.00001.x

