# Supplementary material for: Simulating rewetting events in intermittent rivers and ephemeral streams: A global analysis of leached nutrients and organic matter
Source: Glob Chang Biol. 2019 Feb 25;25(5):1591–611. doi: 10.1111/gcb.14537 (PMC6850495; doi:10.1111/gcb.14537)
Supplement: Supplementary file 1 [file GCB-25-1591-s001.docx]

**Supplementary Material**

**Simulating rewetting events in intermittent rivers and ephemeral streams: a global analysis of leached nutrients and organic matter**

O. Shumilova, D. Zak, T. Datry, D. von Schiller, R. Corti, A. Foulquier, B. Obrador, K. Tockner, F. Altermatt, M. I. Arce, S. Arnon, D. Banas, A. Banegas-Medina, E. Beller, M. L. Blanchette, J. F. Blanco-Libreros, J. Blessing, I. Gonçalves Boëchat, K. Boersma, M. T. Bogan, N. Bonada, N. Bond, K. Brintrup, A. Bruder, R. Burrows, T. Cancellario, S. M. Carlson, S. Cauvy-Fraunié, N. Cid, M. Danger, B. de Freitas Terra, A. M. De Girolamo, R. del Campo, F. Dyer, A. Elosegi, E. Faye, C. Febria, R. Figueroa, B. Four, M. O. Gessner, P. Gnohossou, R. Gómez Cerezo, L. Gómez-Gener, M. A. S. Graça, S. Guareschi, B. Gücker, J. L. Hwan, S. Kubheka, S. D. Langhans, C. Leigh, C. Little, S. Lorenz, J. Marshall, A. McIntosh, C. Mendoza-Lera, E. I. Meyer, M. Miliša, M. C. Mlambo, M. Moleón, P. Negus, D. Niyogi, A. Papatheodoulou, I. Pardo, P. Paril, V. Pešić, P. Rodríguez-Lozano, R. J. Rolls, M. Mar Sánchez-Montoya, A. Savić, A. Steward, R. Stubbington, A. Taleb, R. Vander Vorste, N. Waltham, A. Zoppini and C. Zarfl

**Methods**

*Material collection*

For each river, we selected a reach 10 times as long as the active channel width, and sampled randomly 5% of the riverbed using 1 m^2^ quadrats. From each quadrat, we first collected leaf litter. Then, if present, epilithic biofilm mats deposited over the sediments or growing on rocks were quantitatively sampled in a 20 cm × 20 cm area within each quadrat by removing mats and/or by scraping the surface of stones with a razor blade. Finally, superficial fine sediment (0-10 cm depth, gravel to clay) was collected with a spoon or a shovel. Leaf litter (~60 g), biofilm/dry algae, and sediment (up to 3 L) samples were placed in separate ziplock bags. For each reach, individual subsamples of the same substrate types were pooled.

*Leaching experiments*

After filtration samples for analysis of dissolved organic carbon (DOC) and inorganic nitrogen species (see below) were acidified with 2M HCl to pH 3 - 4 using a dropper. Non-acidified samples for dissolved organic matter (DOM) characterization and quantification with size exclusion chromatography were stored at 2 °C until analysis within two weeks. Preliminary tests have shown that storage under these conditions does not alter DOC concentrations or DOM composition (Heinz & Zak, 2018). Samples for the analysis of ammonium (N-NH_4_^+^), nitrate (N-NO_3_^-^), and optical indices of DOM through absorbance-fluorescence measurements were stored at -20 °C and analyzed within one month. Concentrations of soluble reactive phosphorus (SRP) and phenolics were analyzed immediately after filtration (cellulose-acetate filter, 0.45 µm pore size).

*Physical and chemical characterization of substrates*

Leaves and biofilms were ground to 5 μm with a ball mill (MM301, Retsch GmbH, Haan, Germany) and fractions of carbon and nitrogen (%C and %N, respectively) were estimated in three 10-mg subsamples (FlashEA 1112, Fisher Scientific, Waltham, Massachusetts, USA). The organic C and total N content of sediments were measured after grinding and acidification with 2M HCl (TruSpec Micro CHNS, Leco Corporation, USA), using two analytical replicates. After elimination of organic matter (OM) with H_2_O_2_, sediment texture descriptors (fractions (%) of sand, silt, clay and their mean and median particle size) were determined with a laser-light diffraction instrument (Coulter LS 230, Beckman-Coulter, USA), using one replicate per sediment sample.

*Chemical characterization of leachates*

DOC was measured as nonpurgeable organic carbon in a filtered sample with a total organic carbon (TOC) analyzer (multi N/C 2100, Jena Analytics, Jena, Germany) according to DIN EN 1484 (DEV, H3). SRP was determined with the ammonium molybdate spectrometric method (DIN EN 1189 D11) using a Cary 1E Spectrophotometer (Varian). N-NH_4_^+^ and N-NO_3_^-^ were determined colorimetrically using the photometry CFA method (Skalar SAN, Skalar Analytical B.V., The Netherlands) following the guidelines in EN ISO 11732 (DEV-E 23) and EN ISO 13395 (DEV, D 28), respectively. Concentration of phenolics was determined according to the Folin-Ciocalteau method using a spectrophotometer (SPEKOL 2000, Analytic Jena, Jena, Germany) and expressed in units of gallic acid equivalent (GAE, as described in Box, 1983; Ainsworth & Gillespie, 2007).

Detection limits of the analysis procedures were: DOC 0.5 mg C L^-1^, N-NH_4_^+^ 0.03 mg N L^-1^, N-NO_3_^-^ 0.01 mg N L^-1^, SRP 3 µg P L^-1^, phenolics 0.01 mg GAE L^-1^. When measurement results were lower than the detection limit (less than 15 % of the samples), concentration values were set to half of the detection limit in accordance with the recommendations by USEPA (2000).

*Size exclusion chromatography (SEC)*

The weak cation-exchange chromatographic column of SEC separates molecules according to molecular size and polarity (for details see Huber, Balz, Abert & Pronk, 2011). The sub-categories (biopolymers, humic substances, low-molecular weight substances) were assigned using the customized software programme ChromCALC (DOC-Labor Huber, Karlsruhe, Germany) based on standards of the International Humic Substances Society. The detection limit for each category was 0.01 mg C L^-1^.

*Spectroscopic analysis*

Samples stored for DOM analysis at -20 °C were thawed and acclimated to room temperature. When samples were too concentrated for the optical measurements, evaluated visually and based on measured concentrations of DOC, the samples were diluted with distilled water and the dilution factor was accounted for in further calculations.

Absorbance spectra were measured from 250 to 600 nm with 5-nm steps using a 10-mm quartz cuvette and a scan speed of 12 000 nm min^-1^. Fluorescence EEMs were determined with excitation wavelengths 250-600 nm (5 nm increments) and emission ranges 250-550 nm (1.77 increments).

**References**

Box, J. D. (1983). Investigation of the Folin-Ciocalteu phenol reagent for the determination of poly phenolic substances in natural waters. *Water Research, 17,* 511−525.

Heinz, M., & Zak, D. (2018). Storage effects on quantity and composition of dissolved organic carbon and nitrogen of lake water, leaf leachate and peat soil water. *Water Research, 130,* 98−104.

Huber, S. A., Balz, A., Abert, M., & Pronk, W. (2011). Characterisation of aquatic humic and non-humic matter with size-exclusion chromatography−organic carbon detection−organic nitrogen detection (LC- OCD-OND). *Water Research, 45,* 879−885.

USEPA (U.S. Environmental Protection Agency) (2000). Guidance for Data Quality Assessment: Practical Methods for Data Analysis, EPA QA/G9, QA00/Update. Office of Environmental Information, U. S. Environmental Protection Agency, Washington, DC. Retrieved from https://www.epa.gov/sites/production/files/2015-06/documents/g9-final.pdf. Last acceced 19.07.2018.

**Table S1** Comparison of the environmental variables and substrate characteristics across climate zones (N - number of samples). PET - potential evapotranspiration.

|  | **Arid (N=29)** | | | | **Temperate (N=142)** | | | | **Tropical (N=19)** | | | | | **Continental (N=13)** | | | |
| --- | --- | --- | --- | --- | --- | --- | --- | --- | --- | --- | --- | --- | --- | --- | --- | --- | --- |
| Parameter | Median | Mean±SD | Min | Max | Median | Mean±SD | Min | Max | Median | Mean±SD | | Min | Max | Median | Mean±SD | Min | Max |
| **Dry period (days)** | 190 | 260±241 | 10 | 800 | 73 | 81±53 | 6 | 300 | 120 | 136±74 | | 45 | 300 | 90 | 106±67 | 40 | 243 |
| **Riparian cover (%)** | 40 | 49±33 | 2 | 100 | 75 | 64±33 | 0 | 100 | 60 | 63±27 | | 10 | 100 | 90 | 81±25 | 10 | 100 |
| **River width (m)** | 4.0 | 4.0±2.3 | 0.5 | 10.7 | 3.0 | 3.3±2.3 | 0.4 | 13.5 | 5.0 | 5.6±2.4 | | 2.0 | 10.0 | 2.0 | 2.5±1.5 | 1.2 | 7.0 |
| **Aridity** | 289 | 343±153 | 73 | 682 | 592 | 617±200 | 5 | 1667 | 993 | 1013±180 | | 830 | 1653 | 485 | 529±75 | 461 | 689 |
| **PET** | 343 | 1442±226 | 1081 | 1817 | 977 | 1062±201 | 635 | 1721 | 1590 | 1553±221 | | 1101 | 1896 | 763 | 778±50 | 730 | 910 |
| **% urban areas** | 1 | 3.4±6.6 | 0 | 29 | 0 | 5±12 | 0 | 100 | 5 | 18±23 | | 0 | 80 | 1 | 8±20 | 0 | 80 |
| **% forest areas** | 30 | 36±34 | 0 | 100 | 65 | 59±34 | 0 | 100 | 30 | 46±30 | | 5 | 100 | 21 | 43±37 | 5 | 100 |
| **% pasture areas** | 61 | 59 ±35 | 0 | 100 | 28 | 36±33 | 0 | 100 | 25 | 36±32 | | 0 | 94 | 69 | 49±36 | 0 | 93 |
| **Z (m)** | 195 | 519±809 | 580 | 2852 | 287 | 417±338 | 24 | 1658 | 70 | 249±279 | | 23 | 845 | 80 | 198±185 | 42 | 666 |
| **Sediments** | **N=28** | | | | **N=129** | | | | **N=15** | | | | | **N=12** | | | |
| **%C** | 0.4 | 0.5±0.4 | 0.1 | 1.9 | 0.8 | 1.8±2.0 | 0.1 | 10.4 | 0.2 | 0.6±0.5 | | 0.1 | 1.7 | 1.6 | 2.8±2.7 | 0.4 | 8.5 |
| **%N** | 0.02 | 0.03±0.03 | 0.01 | 0.10 | 0.04 | 0.07±0.10 | 0.01 | 0.70 | 0.02 | 0.04±0.04 | | 0.01 | 0.10 | 0.09 | 0.20±0.20 | 0.03 | 0.50 |
| **C:N** | 17 | 28±30 | 2 | 138 | 17 | 67±1667 | 6 | 1353 | 14 | 15±3 | | 9 | 23 | 15 | 26 ±29 | 9 | 109 |
| **Mean size(mm)** | 67 | 289±304 | 15 | 1019 | 406 | 401 ±334 | 9 | 1209 | 590 | 504±310 | | 33 | 1077 | 304 | 345±222 | 24 | 693 |
| **%Clay** | 6 | 8.0 ±8.0 | 0.3 | 32.0 | 3.5 | 6.0 ±5.5 | 0.2 | 22.0 | 1.8 | 4.8±5.5 | | 0.2 | 16.0 | 3.0 | 5.0±5.0 | 0.9 | 17.0 |
| **% Silt** | 50 | 55.0 ±32.0 | 4.0 | 99.0 | 81.0 | 63.0±33.0 | 0.1 | 99.9 | 93.0 | 74.0 ±29.0 | | 17.0 | 98.0 | 84.0 | 72.0 ±28.0 | 13.0 | 97.0 |
| **Leaves** | **N=27** | | | | **N=138** | | | | **N=19** | | | | | **N=13** | | | |
| **%C** | 42 | 39±10 | 11 | 53 | 44 | 42±8 | 13 | 60 | 42 | 40±6 | | 23 | 48 | 45 | 43±7 | 21 | 50 |
| **%N** | 0.9 | 1.0±0.4 | 0.3 | 2.4 | 1.2 | 1.1±0.4 | 0.1 | 2.5 | 1.3 | 1.3±0.4 | | 0.8 | 2.2 | 1.8 | 1.6±0.4 | 0.8 | 2.1 |
| **C:N** | 41 | 43±14 | 17 | 86 | 35 | 41±10 | 17 | 154 | 34 | 33±20 | | 10 | 50 | 26 | 29±10 | 21 | 58 |
| **Weight,**  **g m^-2^** | 32 | 71±135 | 0.1 | 714 | 36 | 93±147 | 0.4 | 963 | 66 | 168±224 | | 3 | 755 | 54 | 67±45 | 15 | 180 |
| **Biofilms** | **N=4** | | | | **N=33** | | | | **N=3** | | | | | **N=0** | | | |
| **%C** | 10.0 | 21.0±21.0 | 3.7 | 50.0 | 14.0 | 17.0±10.0 | 1.5 | 48.0 | 12.4 | 12.9 | 7.3 | | 19.0 |  |  |  |  |
| **%N** | 0.5 | 0.4±0.3 | 0.1 | 0.8 | 0.7 | 0.9±0.7 | 0.1 | 2.9 | 1.7 | 1.3±0.5 | 0.5 | | 1.7 |  |  |  |  |
| **C:N** | 7 | 45±18 | 21 | 66 | 22 | 26±16 | 3 | 56 | 11 | 11±3 | 7 | | 15 |  |  |  |  |
| **Weight, g m^-2^** | 15.0 | 16.0±17.0 | 0.03 | 35.0 | 5.0 | 35.0±77.0 | 0.3 | 327.0 | 0.3 | 0.4±0.2 | 0.3 | | 0.7 |  |  |  |  |

**Table S2** Total and relative leaching rates of nutrients and organic matter species from leaves, biofilms and bed sediments of IRES globally

|  |  |  | **Leaves** | | | | | **Biofilms** | | | | | **Sediments** | | | | |
| --- | --- | --- | --- | --- | --- | --- | --- | --- | --- | --- | --- | --- | --- | --- | --- | --- | --- |
| Parameter | Unit | Leaching rate | Median | Mean±SD | Min | Max | IQR_d_^*^,  % | Median | Mean±SD | Min | Max | IQR_d_,  % | Median | Mean±SD | Min | Max | IQR_d_,  % |
| **DOC** | mg g^-1^ dry mass | Total | 26.94 | 35.4±  27.0 | 2.6 | 151.7 | 242 | 6.5 | 12.3±  18.2 | 0.1 | 90.2 | 925 | 0.07 | 0.1±  0.2 | 0.0005 | 1.5 | 192 |
|  | mg g^-1^ C | Relative | 66.50 | 86.9±  69.8 | 9.2 | 396.3 | 236 | 49.9 | 73.6±  81.5 | 3.2 | 373.1 | 432 | 13.7 | 18.4±  16.1 | 0.016 | 85.6 | 314 |
| **N-NH_4_^+^** | mg g^-1^ dry mass | Total | 0.08 | 0.10±  0.07 | 0.006 | 0.4 | 173 | 0.04 | 0.05±  0.06 | 0.006 | 0.3 | 300 | 0.002 | 0.004±0.006 | 0.00005 | 0.05 | 304 |
|  | mg g^-1^ N | Relative | 7.23 | 8.49±  5.59 | 0.665 | 33.7 | 143 | 7.87 | 8.1±  5.2 | 0.7 | 17.7 | 204 | 4.6 | 8.3±9.4 | 0.3 | 51.8 | 356 |
| **N-NO_3_^-^** | mg g^-1^ dry mass | Total | 0.002 | 0.01±  0.03 | 0.000002 | 0.2 | 453 | 0.01 | 0.02±  0.02 | 0.0003 | 0.08 | 537 | 0.004 | 0.008±0.012 | 0.00005 | 0.09 | 465 |
|  | mg g^-1^ N | Relative | 0.31 | 1.19±  2.17 | 0.00017 | 15.1 | 608 | 2.6 | 4.7± 6.7 | 0.05 | 32.9 | 802 | 11.4 | 22.0±  33.4 | 0.03 | 271.7 | 535 |
| **DON** | mg g^-1^ dry mass | Total | 0.16 | 0.22±  0.15 | 0.008 | 0.6 | 233 | 0.1 | 0.1±  0.1 | 0.01 | 0.3 | 245 | 0.002 | 0.003±0.003 | 0.000026 | 0.02 | 474 |
|  | mg g^-1^ N | Relative | 15.92 | 20.03±  17.02 | 1.13 | 86.5 | 186 | 11.1 | 16.5±12.2 | 5.3 | 45.8 | 163 | 4.5 | 5.8±  4.3 | 0.3 | 22.6 | 145 |
| **SRP** | mg g^-1^ dry mass | Total | 0.15 | 0.19±  0.14 | 0.02 | 0.8 | 200 | 0.05 | 0.1±0.2 | 0.002 | 0.7 | 1803 | 0.001 | 0.001±0.003 | 0.0000031 | 0.02 | 361 |
| **Phenolics** | mg of GAE* g^-1^ dry mass | Total | 8.96 | 13.56±  13.40 | 0.17 | 59.5 | 459 | 0.22 | 0.4±0.5 | 0.001 | 2.2 | 2650 | 0.005±0.010 | 0.020 | 0.0000025 | 0.19 | 991 |
|  | mg of GAE* g^-1^ of C | Relative | 21.84 | 32.84±  31.75 | 0.59 | 147.3 | 459 | 1.64 | 3.0±4.9 | 0.01 | 27.1 | 1799 | 0.61±1.41 | 2.3 | 0.00015 | 18.36 | 1856 |

* *GAE* – gallic acid equivalent

** IQR_d_ – percentage increase of the value of third quartile versus first quartile of data distribution (variability measure)

**Table S3** Qualitative characteristics of dissolved organic matter in leachates released from leaves, biofilms and bed sediments of IRES globally

|  |  | **Leaves** | | | | **Biofilms** | | | | **Sediments** | | | |
| --- | --- | --- | --- | --- | --- | --- | --- | --- | --- | --- | --- | --- | --- |
| Parameter | Unit | Median | Mean±SD | Min | Max | Median | Mean±SD | Min | Max | Median | Mean±SD | Min | Max |
| **SUVA_254_** | mg C L^-1^ | 1.59 | 1.74±0.76 | 0.32 | 4.51 | 0.71 | 0.81±0.36 | 0.13 | 1.61 | 1.75 | 1.73±0.60 | 0.37 | 3.19 |
| **DOC:DON** | - | 125.8 | 152.4±99.8 | 28.36 | 501.33 | 40.18 | 116.86±240.77 | 20.55 | 158.34 | 38.52 | 49.72±28.20 | 14.75 | 148.31 |
| **LMWS** | % | 38.00 | 39.06±11.64 | 18.00 | 66.00 | 16.50 | 20.67±15.89 | 0.20 | 36.70 | 19.00 | 22.54±13.33 | 1.4 | 80.0 |
| **BP** | % | 4.20 | 5.25±4.20 | 0.50 | 19.20 | 23.40 | 22.02±11.51 | 29.00 | 70.00 | 10.00 | 11.45±5.76 | 2.3 | 34.0 |
| **HS** | % | 55.30 | 55.58±10.90 | 32.00 | 78.70 | 56.00 | 56.69±10.33 | 8.00 | 67.00 | 69.00 | 65.75±11.30 | 18.0 | 84.0 |
| **Phenolics:**  **DOC** | - | 0.33 | 0.34±0.18 | 0.019 | 0.903 | 0.03 | 0.03±0.03 | 0.00035 | 0.12 | 0.06 | 0.07±0.07 | 0.00005 | 0.50 |
| **FI** | - | 1.36 | 1.40±0.26 | 0.60 | 2.67 | 1.49 | 1.49±0.33 | 0.60 | 2.24 | 1.38 | 1.50±0.42 | 0.96 | 3.51 |
| **HIX** | - | 0.57 | 0.81±0.81 | 0.04 | 6.57 | 1.15 | 1.40±0.85 | 0.42 | 3.58 | 2.69 | 2.86±1.60 | 0.28 | 7.84 |
| **β:α** | - | 0.41 | 0.44±0.21 | 0.15 | 1.28 | 0.63 | 0.65±0.24 | 0.10 | 1.28 | 0.58 | 0.60±0.21 | 0.07 | 1.59 |

**Table S4** Results of the Kruskal-Wallis test followed by Dunn test for post-hoc comparison for the total and relative leaching rates of nutrients and organic matter species from leaves and bed sediments in IRES across different climate zones (Abbreviations used for climate zones: A - arid, Temp – temperate, Trop – tropical, C – continental; n.s. – not significant)

|  |  |  | **Leaves** | | | | **Sediment** | | | |
| --- | --- | --- | --- | --- | --- | --- | --- | --- | --- | --- |
| **Parameter** | **Unit** | **Leaching rate** | **χ^2^** | **df** | **p-value** | **Dunn test** | **χ^2^** | **df** | **p-value** | **Dunn test** |
| **DOC** | mg g^-1^ dry mass | Total | 7.96 | 3 | 0.047 | n.s. | 22.87 | 3 | <0.0001 | (C, A), p<0.0001;  (C, Temp), p<0.0001;  (C, Trop), p=0.001 |
|  | mg g^-1^ C | Relative | 10.31 | 3 | 0.0160 | n.s. | 6.2 | 3 | 0.102 | n.s. |
| **N-NH_4_^+^** | mg g^-1^ dry mass | Total | 19.04 | 3 | <0.0001 | (C, A), p<0.001;  (C, Temp), p<0.001 | 15.80 | 3 | 0.001 | (C, A), p<0.0001;  (C, Temp), p=0.002 |
|  | mg g^-1^ N | Relative | 6.37 | 3 | 0.09 | n.s. | 2.03 | 3 | 0.57 | n.s. |
| **N-NO_3_^-^** | mg g^-1^ dry mass | Total | 5.90 | 3 | 0.12 | n.s. | 10.58 | 3 | 0.014 | (C,A), p=0.002 |
|  | mg g^-1^ N | Relative | 5.22 | 3 | 0.16 | n.s. | 1.78 | 3 | 0.6199 | n.s. |
| **DON** | mg g^-1^ dry mass | Total | 5.88 | 3 | 0.12 | n.s. | 18.7 | 3 | <0.0001* | (C,A), p<0.0001;  (C, Temp), p=0.006;  (C, Trop), p=0.002;  (A, Temp), p=0.007 |
|  | mg g^-1^ N | Relative | 2.95 | 3 | 0.4 | n.s. | 5.37 | 3 | 0.15 | n.s. |
| **PO_4_^3-^** | mg g^-1^ dry mass | Total | 4.52 | 3 | 0.21 | n.s. | 5.43 | 3 | 0.0002 | (C,A), p<0.0001;  (C, Temp), p<0.0001;  (C, Trop), p=0.003 |
| **Phenolics** | mg of GAE* g^-1^ dry mass | Total | 5.90 | 3 | 0.12 | n.s. | 5.43 | 3 | 0.143 | n.s. |
|  | mg of GAE* g^-1^ of C | Relative | 7.48 | 3 | 0.05 | n.s. | 1.659 | 3 | 0.646 | n.s. |
| **SUVA_254_** | mg C L^-1^ | - | 3.82 | 3 | 0.28 | n.s. | 9.99 | 3 | 0.0018 | (C,A), p=0.003 |

**Figure S1** Geographical distribution of areal fluxes of nutrients and organic matter species, g m^-2^ (intervals selected based on the equal numbers of samples)


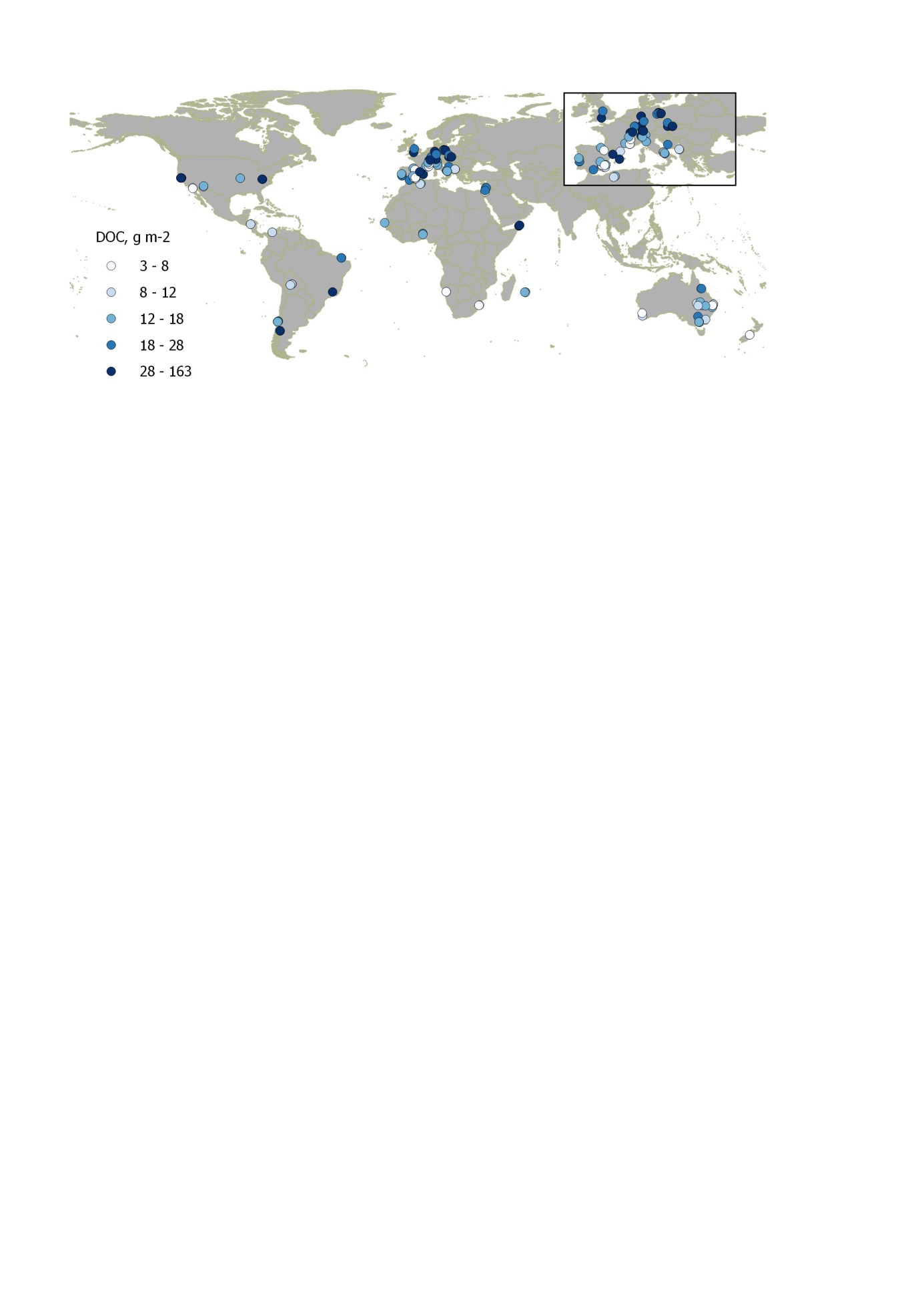


**A**


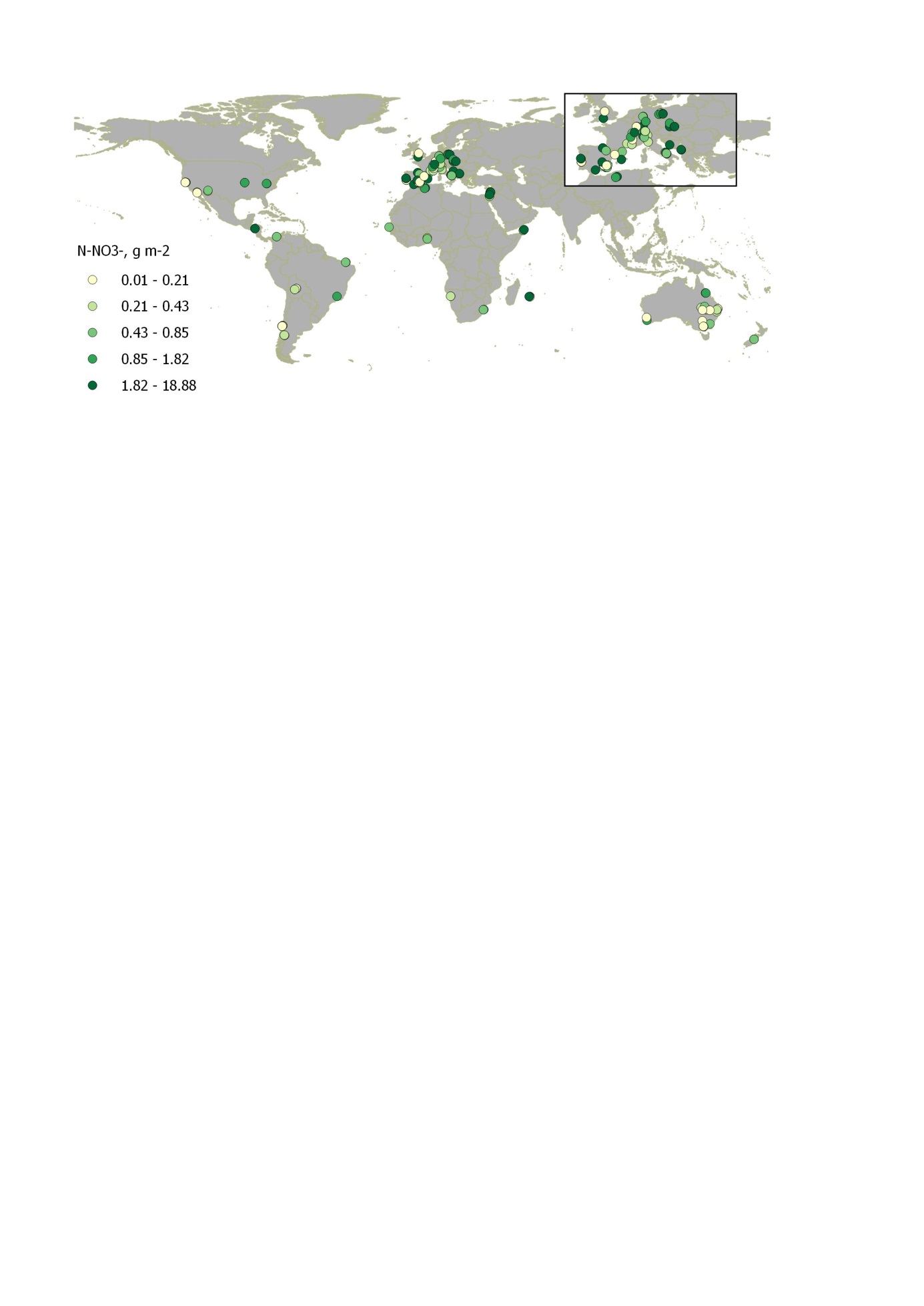


**B**


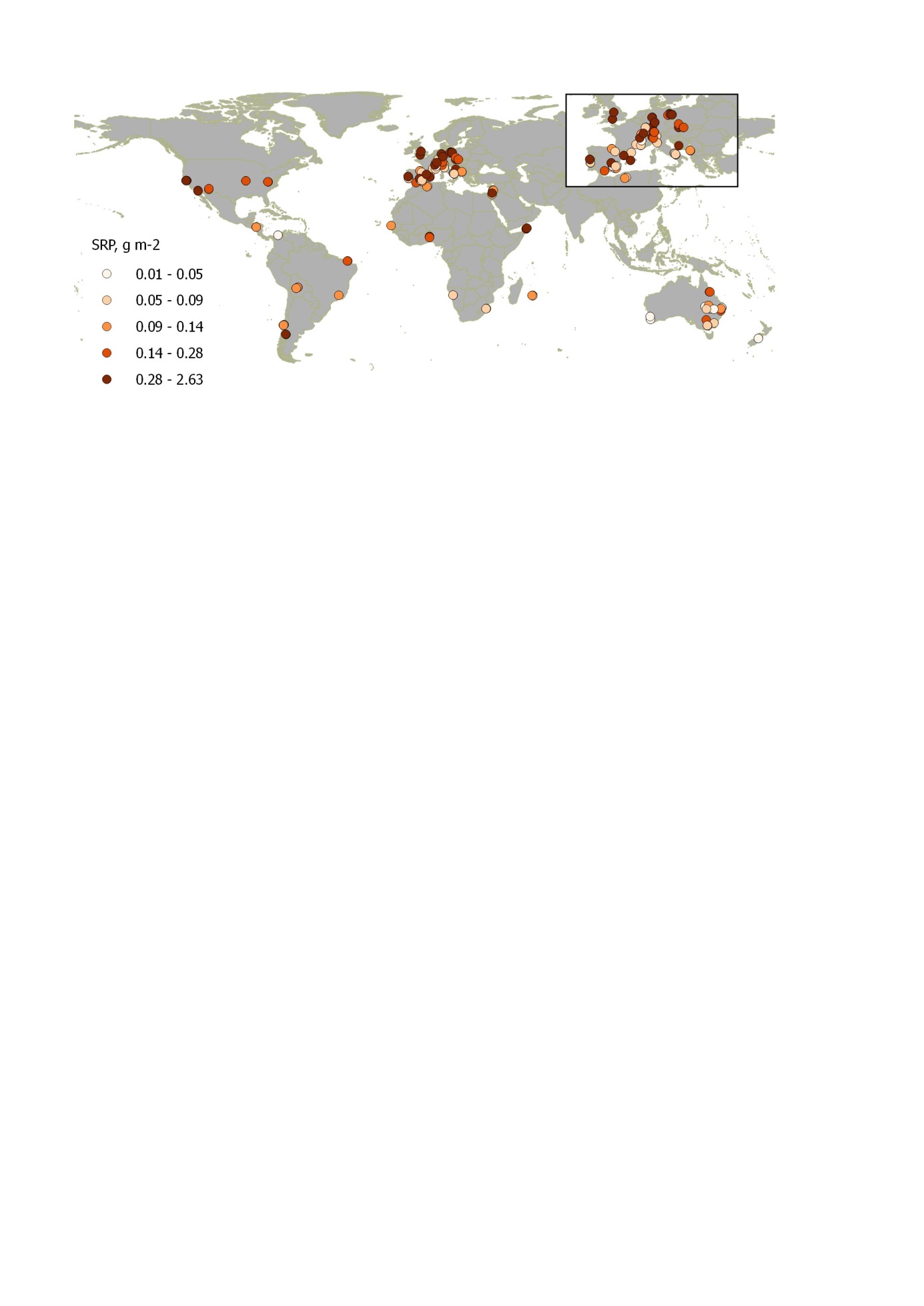


**C**


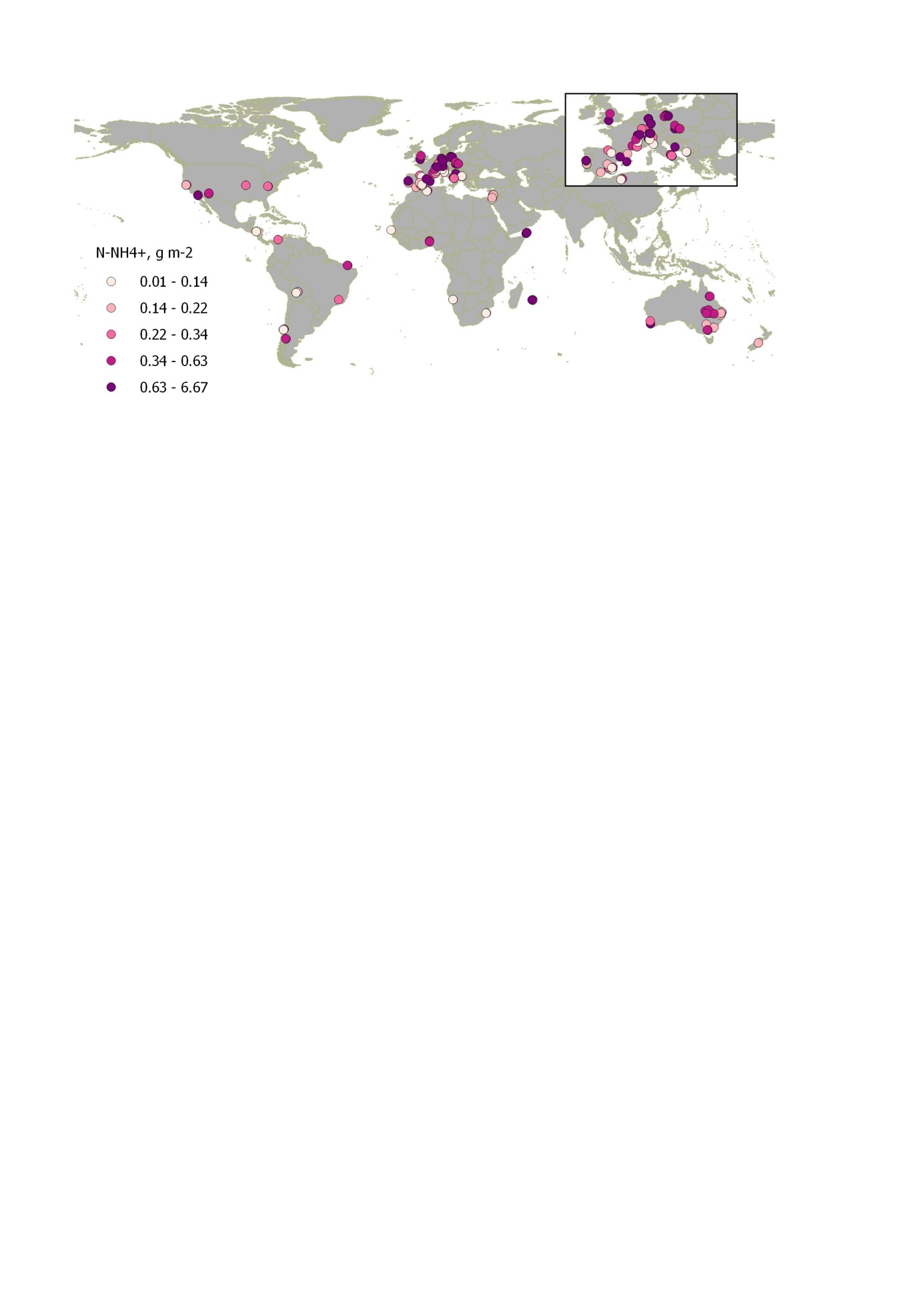


**D**


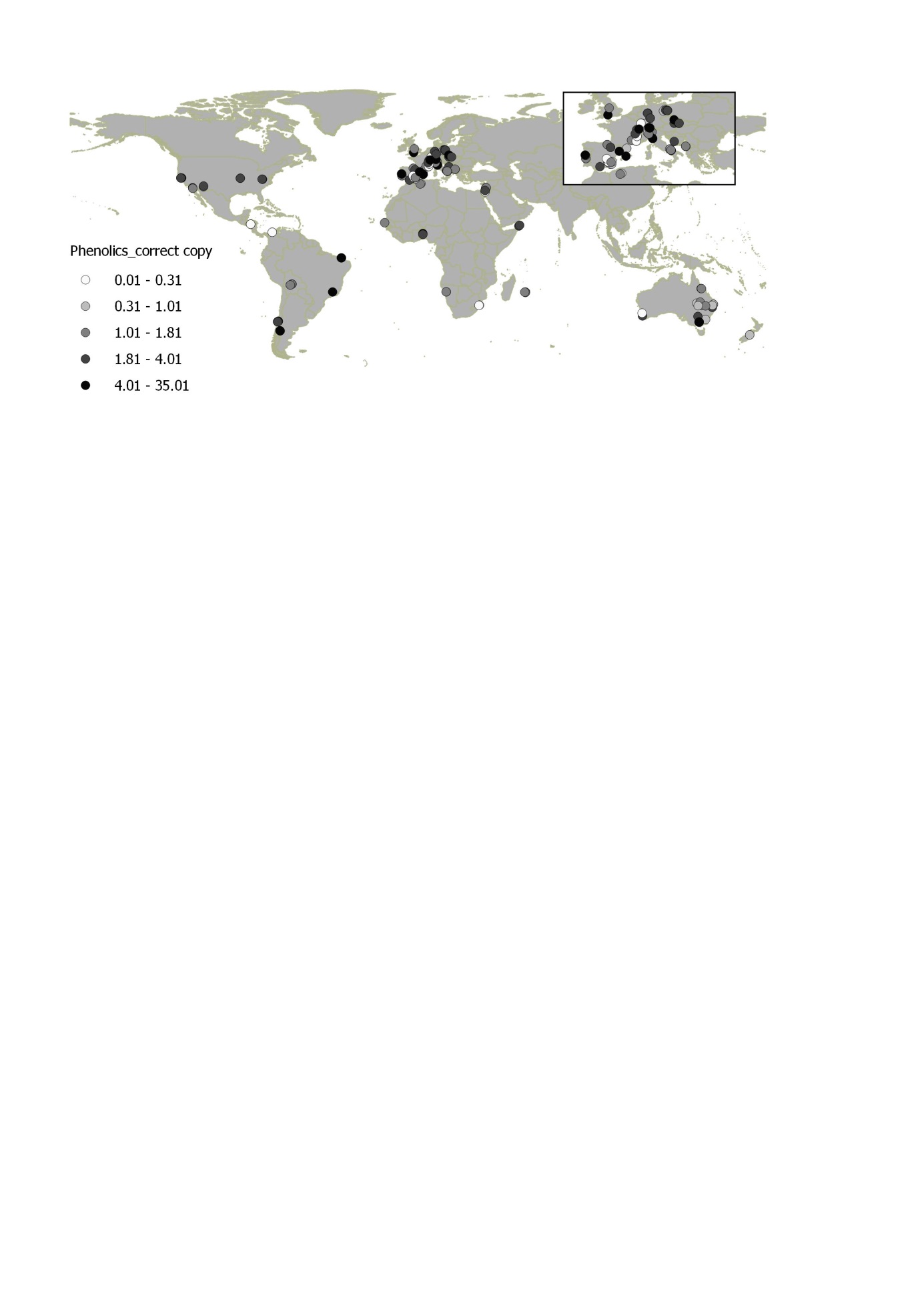


**E**


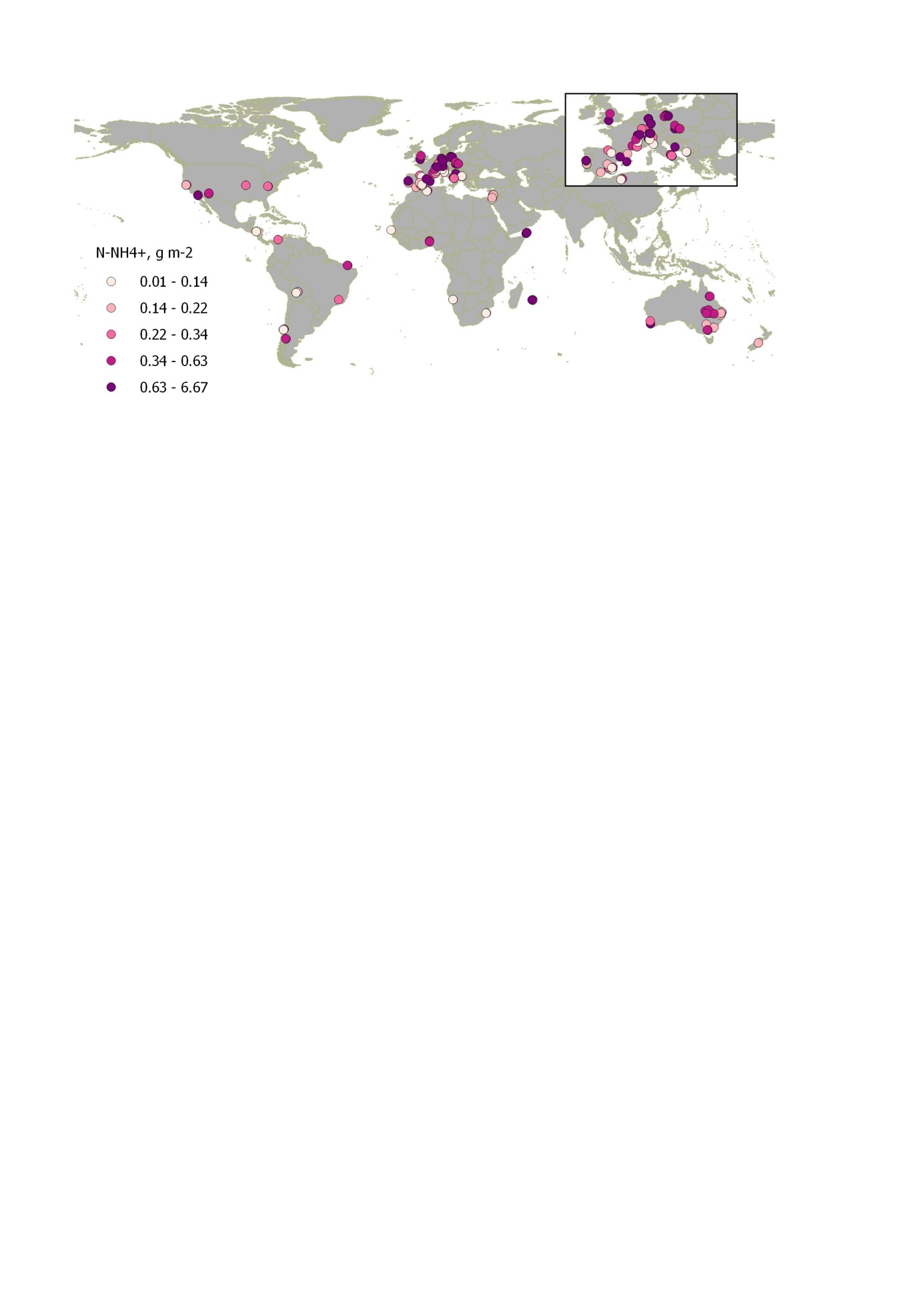


**Table S5** Comparison of nutrient and dissolved organic matter fluxes from IRES in this study and other ecosystems

| **System** | **Nutrient flux** | **Reference** |
| --- | --- | --- |
|  | **DOC flux (g m^−2^ year^−1^)** |  |
| Forested watersheds with no wetland component (N=30), eight states in the eastern United States | 0.5 – 5.7 (min - max) | Table 1 in Raymond and Saiers, 2010 |
| Temperate forest (NA, NZ, R)* (N=52) | 1.8 (0.34 – 41.7) (med (min – max)) | Table 3 in Hope et al., 1994 |
| Boreal forest (NA, NZ, R) (N=6) | 7.64 (2.53 – 48.38) (med (min – max)) |  |
| Temperate grassland (NA, NZ, R) (N=3) | 0.2 (0.16 – 0.5) (med (min – max)) |  |
| Wetlands (NA, NZ, R) (N=5) | 5.28 (0.18 –14.2) (med (min – max)) |  |
| Temperate forest (Europe) (N=5) | 3.83 (1.04 – 5.26) (med (min – max)) |  |
| Boreal forest (Europe) (N=7) | 4.47 (1.04 – 8.75) (med (min – max)) |  |
| Temperate moorland/ grassland (Europe) (N=8) | 4.32 (0.76 – 8.98) (med (min – max)) |  |
|  | **Mean DOC flux in rainfall** **(g m^−2^ year^−1^)** |  |
| Rio Negro, pristine forested area in the central Amazon | 2.75 | Ward et al., 2017 |
| Paragominas, a heavily deforested area in the eastern Amazon | 12.34 |  |
|  | **Mean throughfall DOC fluxes** **(g m^−2^ year^−1^)** |  |
| Amazon | 6.84 – 19.81 | Ward et al., 2017 |
|  | **Areal flux of DOC per flush event (g m^−2^): median (min – max)** |  |
| IRES in arid zone (N=23) | 9.4 (2.96 – 26.71) | This study |
| IRES in temperate zone (N=105) | 16.7 (3.0 – 162.7) |  |
| IRES in tropical zone (N=15) | 15.9 (3.71 – 28.01) |  |
| IRES in continental zone (N=12) | 43.8 (15.04 – 82.58) |  |
|  | **DIN annual yield (N-NH_4_^+^+N-NO_3_^-^),**  **(g m^−2^ year^−1^): min - max** |  |
| Forested streams in Mediterranean catchment (N=21) | 0.004 – 0.1 | Bernal et al., 2005 |
| Forested streams in temperate catchments in Europe, North America, South Africa (N=70) | 0 – 0.59 | Bernal et al., 2005 |
|  | **Areal flux of DIN (N-NH_4_^+^+N-NO_3_^-^) per flush event (g m^−2^):**  **median (min – max)** |  |
| IRES in arid zone (N=23) | 0.63 (0.04 – 6.00) | This study |
| IRES in temperate zone (N=105) | 0.87 (0.02 – 25.54) |  |
| IRES in tropical zone (N=15) | 1.11(0.20 – 6.65) |  |
| IRES in continental zone (N=12) | 2.26 (0.46 – 12.55) |  |
|  | **Total P load** **(g m^−2^ year^−1^)** |  |
| Kouris streams, Cyprus (N=3) | 0.0062 | Tzoraki et al., 2014 |
| Axios River, Greece | 0.064 | Nikolaidis et al., 2009 |
| La Tordera catchment, North East Spain | 0.081 | Gaille et al., 2012 |
|  | **Areal flux of SRP per flush event (g m^−2^): median (min – max)** |  |
| IRES in arid zone (N=23) | 0.07 (0.03 – 0.57) | This study |
| IRES in temperate zone (N=105) | 0.10 (0.02 – 2.63) |  |
| IRES in tropical zone (N=15) | 0.11 (0.03 – 0.51) |  |
| IRES in continental zone (N=12) | 0.36 (0.15 – 1.48) |  |

* Abbreviations used: NA – North America, NZ –New Zeeland, R - Russia

**References**

Bernal, S., Butturini, A. & Sabater, F. (2005). Seasonal variations of dissolved nitrogen and DOC:DON ratios in an intermittent Mediterranean stream. *Biogeochemistry, 75*, 351-372.

Gaille, F., Riera, J. L. et al (2012). Modelling nitrogen and phosphorus loads in a Mediterranean river catchment (La Tordera, NE Spain). *Hydrology and Earth System Science, 16,* 2417–2435.

Hope, D., Billett, M. F. & Cresser, M.S. (1994). A review of the export of carbon in river water: fluxes and processes. *Environmental Pollution, 84,* 301-324.

Nikolaidis, N. P., Karageorgis, A.P., Kapsimalis, V., Drakopouloub, P., Skoulikidis, N., Behrend, N., Levkov, Z. (2009). Management of nutrient emissions of Axios River catchment: their effect in the coastal zone of Thermaikos Gulf, Greece. *Ecological Modelling, 220*, 383–396.

Raymond, R., & Saiers, J. E. (2010). Event controlled DOC export from forested watersheds. *Biogeochemistry, 100,* 197–209.

Tzoraki, O., Cooper, D., Dörflinger, G., & Panagos, A. (2014). A new MONERIS in-stream retention module to account nutrient budget of a temporary tiver in Cyprus. *Water Resources Management, 28*, 2917 – 2935.

Ward, N. D., Bianchi, T.S., Medeiros, P. M., Seidel, M., Richey, J. Y., Keil, R.G., & Sawakuchi, H. O. (2017). Where carbon goes when water flows: carbon cycling across the aquatic continuum. *Frontiers in Marine Science, 4*, 1 - 27.
